# TAZ interactome analysis using nanotrap-based affinity purification–mass spectrometry

**DOI:** 10.1242/jcs.263527

**Published:** 2025-02-24

**Authors:** Jonathan Kelebeev, Anastasia MacKeracher, Tetsuaki Miyake, John C. McDermott

**Affiliations:** ^1^ Department of Biology, York University, Toronto, ON, M3J 1P3, Canada; ^2^ Muscle Health Research Centre (MHRC), York University, Toronto, ON, M3J 1P3, Canada; ^3^ Centre for Research in Biomolecular Interactions (CRBI), York University, Toronto, ON, M3J 1P3, Canada

**Keywords:** Nanobodies, Myogenesis, CARM1, Interactome

## Abstract

Characterization of protein–protein interactions (PPIs) is a fundamental goal in the post-genomic era. Here, we document a generally applicable approach to identify cellular protein interactomes using a combination of nanobody-based affinity purification (AP) coupled with liquid chromatography and tandem mass spectrometry (LC–MS/MS). The Hippo signaling regulator TAZ (also known as WWTR1) functions as a transcriptional co-repressor or activator depending on its PPI network; we therefore undertook an unbiased proteomic screen to identify TAZ PPIs in striated muscle cells. A GFP nanotrap-based AP approach coupled with protein identification through LC–MS/MS was used to document a comprehensive list of known and novel TAZ interactome components. Informatic analysis of the interactome documented known components of the Hippo signaling pathway and multiple epigenetic regulators such as the NuRD, FACT and SWI/SNF complexes and the pro-myogenic CARM1 methyltransferase. Hippo pathway reporter gene (HOP/HIP) analysis indicated that CARM1 represses TAZ transcriptional co-activator function, promoting TAZ Ser89 phosphorylation and TAZ cytoplasmic sequestration. MS analysis revealed that CARM1 dimethylates TAZ at Arg77 in a PGPR*LAGG consensus peptide, resulting in enhanced TAZ Ser89 phosphorylation. These studies underline the utility of a nanobody-based AP approach for interactome analysis.

## Teaser

**Highlighted Article:** A robust, generically applicable nanobody-based affinity purification–mass spectrometry approach is described to identify the TAZ protein interactome in mammalian cells.

## INTRODUCTION

Understanding the landscape of protein–protein interactions (PPIs) is a key feature of the post-genomic era. Dissecting and characterizing the complexity of these interactions has proved a formidable task, requiring a flexible toolbox of biochemical and genetic approaches. The progressive exponential development of mass spectrometry (MS)-based techniques in protein identification and quantitation is at the forefront of protein interaction analysis. Here, we document a generally applicable approach to robustly identify protein interactomes using a combination of nanobody-based affinity purification (AP) coupled with liquid chromatography and tandem MS (AP–LC–MS/MS).

The particular biological system that we employed in this study was the evolutionarily conserved Hippo signaling pathway, which functions as a central regulator of organ growth and size by controlling cell proliferation and apoptosis (Ma et al., 2019). At the molecular level, a canonical Hippo signaling pathway has been elucidated in which cell proliferation is restricted by LATS1/2-mediated phosphorylation of the downstream transcriptional effectors Yes-associated protein 1 (YAP1, also known as YAP) and transcriptional co-activator with PDZ-binding motif (TAZ, also known as WWTR1) at conserved serine residues, including Ser127 in YAP and Ser89 in TAZ (Basu et al., 2003; Zhao et al., 2007). Phosphorylated YAP and TAZ (hereafter YAP/TAZ) are sequestered in the cytoplasm in complexes with 14-3-3 proteins and directed for proteasome-mediated degradation (Kanai et al., 2000; Zhao et al., 2007; Tripathi et al., 2022). Conversely, when Hippo signaling is reduced or absent, YAP/TAZ remain unphosphorylated at the conserved phosphorylatable residues and translocate to the nucleus, where they regulate gene expression in concert with DNA-binding transcription factors. At present, the most widely documented YAP/TAZ nuclear interactions are with members of the TEAD transcription factor family and some known epigenetic regulators (Murakami et al., 2005; Chan et al., 2009; Zhang et al., 2009; Chang et al., 2018a).

Although YAP and TAZ are often grouped together as Hippo effectors, there are notable differences in their properties and biological roles (Reggiani et al., 2021). Moreover, the capability of TAZ to function as either a potent co-activator or co-repressor of transcription depending on the context prompted us to speculate that TAZ function is largely determined by its PPI network. To address this idea, we undertook a proteomic-based study aimed at identifying the TAZ interactome in striated muscle cells. Using a novel nanotrap-based AP approach coupled with protein identification through LC–MS/MS, we document a comprehensive list of both known and novel TAZ interactome components in myogenic cells. Subsequently, we focused on TAZ interaction with the methyltransferase CARM1, elucidating its role in facilitating TAZ methylation and counteracting its co-repressor function in muscle cells. Collectively, our study documents the TAZ interactome in striated muscle and, more generally, highlights the efficacy of a nanobody-based AP strategy for protein interactome characterization.

## RESULTS

### AP–LC–MS/MS analysis of the TAZ interactome in striated muscle cells

**Fig. 1.**
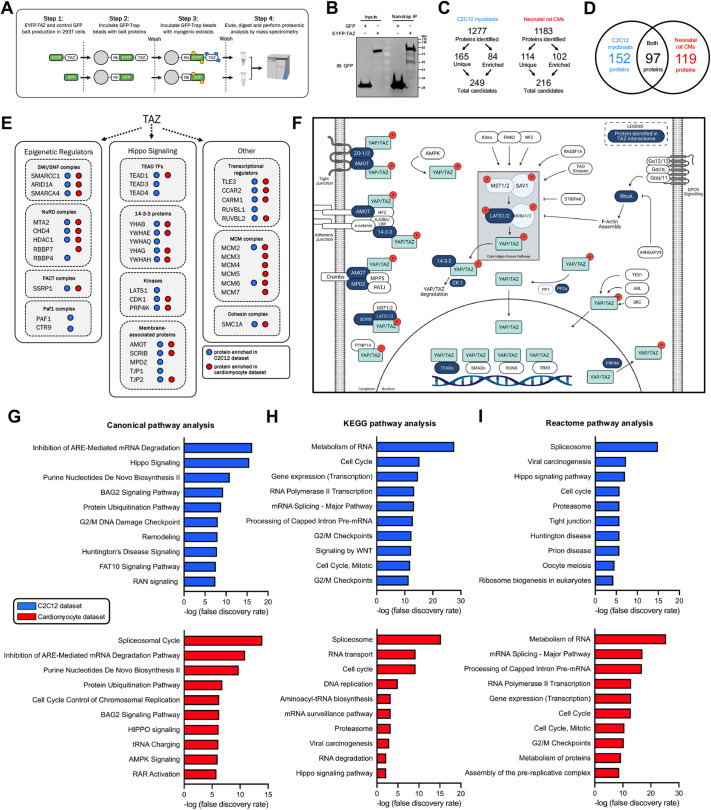
**Interactome study of TAZ in striated muscle.** (A) Schematic overview of the GFP nanotrap-based co-immunoprecipitation method to identify TAZ protein partners in nuclear-enriched C2C12 and whole-cell neonatal rat cardiomyocyte (NRCM) extracts. Two technical replicates were performed for EYFP–TAZ and control GFP samples in both C2C12 and cardiomyocyte experiments. Nb, nanobody. (B) Western blot analysis confirming immunoprecipitation (IP) of GFP bait proteins by GFP-Trap co-immunoprecipitation in HEK293T cells. (C) Flow chart indicating the number of total and enriched TAZ protein partner candidates identified in the C2C12 myoblast and NRCM datasets. (D) Venn diagram indicating the number of unique and overlapping TAZ protein partner candidates between the C2C12 myoblast and NRCM datasets. (E) Summary of notable TAZ protein partners identified in the C2C12 and NRCM extracts. Blue circles indicate proteins enriched in the C2C12 dataset, whereas red circles indicate enrichment in the NRCM dataset. TF, transcription factor. (F) Schematic showing proteins (colored in dark blue) representing known components of the Hippo signaling pathway that were identified in the C2C12 TAZ interactome. (G) Top ten canonical pathways identified by the QIAGEN Ingenuity Pathway Analysis software associated with the C2C12 (blue) and NRCM (red) TAZ interactomes. (H) Top ten KEGG pathways associated with the C2C12 (blue) and NRCM (red) TAZ interactomes. (I) Top ten Reactome pathways associated with the C2C12 (blue) and NRCM (red) TAZ interactomes (see Tables S1–S6 for further documentation of bioinformatic analysis).

TAZ is a potent co-regulator of gene transcription despite its lack of direct DNA-binding activity. Thus, TAZ function and target gene selectivity are determined by PPIs with sequence-specific DNA-binding factors and other transcription complex components (Kim et al., 2018). Therefore, we reasoned that identifying TAZ-interacting partners would further our understanding of the transcriptional mechanisms by which TAZ operates in the nucleus. Based on our previous identification of TAZ as a novel repressor of skeletal muscle gene expression (Tripathi et al., 2022), our initial aim was to characterize the TAZ interactome in skeletal myogenic cells and cardiomyocytes. For this purpose, we used a proteomic strategy utilizing a robust nanotrap technology that we have developed to capture TAZ-interacting proteins from cellular lysates. Initially, we immobilized the TAZ ‘bait’ protein (EYFP–TAZ) and the corresponding EYFP-alone control on GFP nanotrap beads conjugated with GFP-binding protein (GBP) nanobodies. These baits were initially generated by transfection in HEK293T cells. Following wash steps, the bait proteins coupled with GBP beads were incubated with myogenic cell extracts (derived from nuclear-enriched C2C12 or neonatal rat cardiomyocyte extracts). The advantage of this affinity-capture method is that, unlike other proteomic interactome workflows, there is no ectopic expression of the bait protein in the cells of interest, which can lead to stoichiometry issues, alteration of the cellular proteome and non-physiological interactions due to overexpression. This approach thus maintains the unaltered and native state of the endogenous myogenic proteome. In addition, this approach, which obviates the need for transfection of the bait protein, can be used for interactome analysis in cellular model systems that are hard to transfect, such as primary cells (e.g. cardiomyocytes). The workflow of the AP–LC–MS/MS approach is illustrated in Fig. 1A. Efficient expression and subsequent complex formation of EYFP-tagged bait proteins with the GFP nanotrap was confirmed by immunoprecipitation analysis as shown in Fig. 1B. Datasets were processed by filtering out non-specific interactions with less than threefold enrichment (EYFP–TAZ_spectra_/GFP_spectra_, i.e. the MS-derived peptide abundance scores for the designated interacting protein in the EYFP–TAZ precipitate divided by the abundance score of the interacting protein in the GFP control), a threshold that was selected post hoc based on the identification of known, well-characterized TAZ-interacting proteins. Unique proteins that co-immunoprecipitated with EYFP–TAZ that were not found to co-immunoprecipitate with the EYFP control were automatically included in the list of candidates. After processing, the C2C12 and cardiomyocyte datasets featured 249 and 216 protein candidates, respectively (Fig. 1C), whereas 97 common proteins were identified in both the C2C12 and cardiomyocyte datasets (Fig. 1D; Fig. S1). A summary of selected proteins identified from the C2C12 (blue circles) and cardiomyocyte (red circles) interactome lists are shown in Fig. 1E, notably featuring well-known TAZ interactions with components of the SWI/SNF complex (SMARCC1, SMARCA4 and ARID1A) (Chang et al., 2018a), membrane-associated proteins (AMOT, SCRIB and MPDZ) (Chan et al., 2011; Cordenonsi et al., 2011) and components of the Hippo pathway (TEAD transcription factors, 14-3-3 proteins and LATS1 kinase) (Kanai et al., 2000; Lei et al., 2008; Zhang et al., 2009). Novel candidates found within the interactomes encompassed additional components from chromatin-remodeling complexes, such as the FACT complex subunit SSRP1 and NuRD complex subunits (MTA2, CHD4 and HDAC1). Transcriptional regulators, including the co-repressor TLE3, and the arginine methyltransferase CARM1 were also identified (Fig. 1E). A schematic placing the proteins identified in our screen within the broader context of the Hippo signaling pathway is shown in Fig. 1F. Bioinformatic analysis of the C2C12 (blue) and cardiomyocyte (red) datasets was conducted to identify biological processes or pathways pertinent to the proteins interacting with TAZ. Ingenuity pathway analysis revealed Hippo signaling among the top ten associated pathways (Fig. 1G) in both datasets, highlighting the fidelity of the approach to capture well-characterized proteins found within the canonical Hippo signaling pathway. In addition, notable connections were also made with 14-3-3-mediated signaling, Wnt/β-catenin signaling, tight junction signaling, and DNA methylation and transcriptional repression signaling. KEGG and Reactome pathway analysis was also carried out to gather additional insights that might direct further experimentation. The Hippo signaling pathway was again identified by KEGG pathway analysis (Fig. 1H) and the Wnt signaling pathway was identified by Reactome pathway analysis (Fig. 1I).

### Biochemical validation of the TAZ interactome

**Fig. 2.**
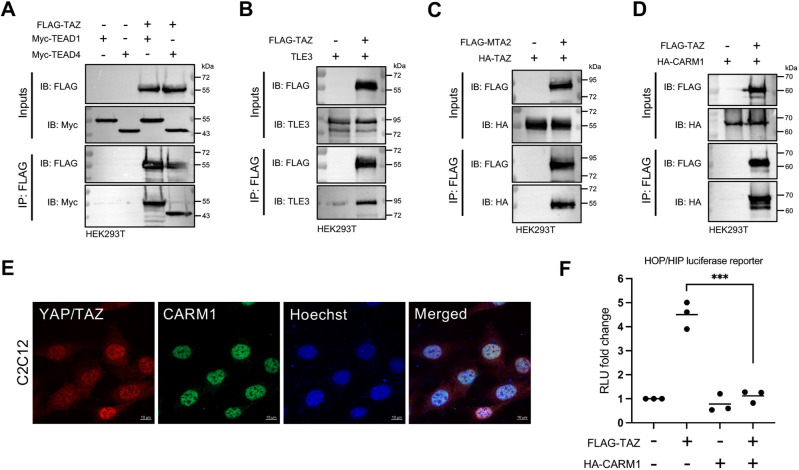
**Biochemical validation of the TAZ interactome.** (A) HEK293T cells were transfected with a Myc–TEAD1 or Myc–TEAD4 plasmid with or without the FLAG–TAZ plasmid and subjected to FLAG immunoprecipitation (IP) with anti-FLAG beads. Eluates were analyzed using immunoblotting (IB) for the contents of co-immunoprecipitated Myc–TEAD1 and Myc–TEAD4 to serve as positive controls for the co-immunoprecipitation analysis. (B) HEK293T cells were transfected with a TLE3 plasmid with or without the FLAG–TAZ plasmid and subjected to FLAG immunoprecipitation with anti-FLAG beads. Eluates were analyzed using immunoblotting for the contents of co-immunoprecipitation with FLAG–TAZ. (C) HEK293T cells were transfected with a HA–TAZ plasmid with or without the FLAG–MTA2 plasmid and subjected to FLAG immunoprecipitation with anti-FLAG beads. Eluates were analyzed using immunoblotting for the contents of co-immunoprecipitation with FLAG–MTA2. (D) HEK293T cells were transfected with a HA–CARM1 plasmid with or without the FLAG–TAZ plasmid and subjected to FLAG immunoprecipitation with anti-FLAG beads. Eluates were analyzed using immunoblotting for the contents of co-immunoprecipitation with FLAG–TAZ. Blots are representative of more than three independent experiments. (E) Immunofluorescence analysis of C2C12 cells during myoblast [cultured in growth medium (GM)] stage immunostained for YAP/TAZ (red) and CARM1 (green) and counterstained with Hoechst 33342 to indicate nuclei (blue). Images are representative of more than three independent experiments. (F) FLAG–TAZ alone and combined with HA–CARM1 was ectopically expressed with the HOP luciferase reporter gene (normalized to the HIP reporter as a negative control). Renilla luciferase served as a transfection control. Transfection with an empty vector (pcDNA3) served as a control for endogenous activity. Triplicate intra-experimental samples were analyzed and the mean of these triplicate technical replicates constituted one biological replicate. Each experiment was repeated three times and the data points on the graphs indicate the biological replicates (*n*=3). Statistical analysis was conducted on PRISM from GraphPad 10.0. Using this software, an unpaired one-tailed *t*-test was used to test for statistical significance. Adjusted *P*-value (***P≤0.001) is indicated for significance compared to the relevant control condition. RLU, relative light units.

To further validate some novel PPIs identified in the interactomes biochemically, we conducted FLAG co-immunoprecipitation experiments between TAZ and several identified interactors in HEK293T cells. Positive controls were established using the known interaction between TAZ and the transcription factors TEAD1 and TEAD4 (Fig. 2A). Among the list of proteins identified in the interactome, we first validated TAZ interaction with the co-repressor TLE3 and the NuRD complex component MTA2 (Fig. 2B,C). These biochemical co-precipitations provided further validation of the efficacy of the GBP nanotrap approach coupled with MS in identifying interactions with relatively low-abundance proteins expressed at normal endogenous levels. Based on our interest in understanding the role of TAZ in myogenic cells, we opted to further concentrate on the interaction with CARM1, given its documented role in myogenesis through interactions with transcription factors such as PAX7 and MEF2C (Chen et al., 2002; Kawabe et al., 2012). As with TLE3 and MTA2, the CARM1–TAZ interaction was confirmed biochemically (Fig. 2D) and localization studies by immunofluorescence revealed that endogenous CARM1 and YAP/TAZ proteins were each localized in the nucleus of C2C12 myoblasts (Fig. 2E). Subsequently, we investigated whether CARM1 influences the transcriptional co-activator properties of TAZ on a TEAD-responsive HOP/HIP luciferase reporter gene assay. We observed that CARM1 expression significantly decreased the potent TAZ-driven activation of HOP/HIP in HEK293T cells (Fig. 2F). These findings suggest that CARM1 antagonizes TAZ co-activator function on a TEAD-responsive reporter gene.

### CARM1 antagonizes TAZ-mediated repression of the myogenic program

**Fig. 3.**
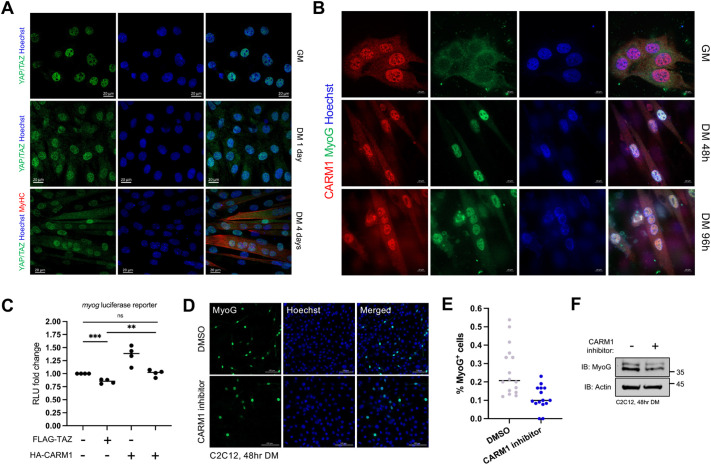
**CARM1 antagonizes TAZ-mediated repression of the myogenic program.** (A) Immunofluorescence analysis of endogenous YAP/TAZ in C2C12 cells during myoblast (culture in GM), early differentiation [culture in differentiation medium (DM) for 1 day] and late differentiation (culture in DM for 4 days) stages. Cells cultured in DM for 4 days were immunostained for MyHC (red) to label myotubes and counterstained with Hoechst 33342 to indicate nuclei (blue). (B) Immunofluorescence analysis of endogenous CARM1 in C2C12 cells during myoblast (GM), early differentiation (DM 48 h), and late differentiation (DM 96 h) stages. Cells were immunostained for MyoG (green) to label cells entering myogenesis and counterstained with Hoechst 33342 to indicate nuclei (blue). Images in A,B are representative of three independent experiments. (C) FLAG–TAZ alone and combined with HA–CARM1 was ectopically expressed with a myogenin promoter reporter gene in C2C12 cells cultured in DM for 48 h. Renilla luciferase served as a transfection control. Transfection with an empty vector (pcDNA3) served as a control for endogenous activity. Triplicate intra-experimental samples were analyzed and the mean of these triplicate technical replicates constituted one biological replicate. Each experiment was repeated four times and the data points on the graphs indicate the biological replicates (*n*=4). Each point represents an average of three technical replicates from an individual biological sample. Unpaired one-tailed *t*-test was utilized to test for statistical significance. Adjusted *P*-values (**P≤0.01, ***P≤0.001) are indicated for significance compared to the relevant control condition. ns, not significant. (D) C2C12 cells were cultured in DM with 5 μM CARM1 inhibitor for 24 h, followed by a 24-h recovery period in DM. Cells were then immunostained for MyoG (green) and counterstained with Hoechst 33342 to visualize nuclei (blue). C2C12 cells treated with DMSO served as the control. (E) Quantification of the fraction of MyoG^+^ cells in DMSO- and CARM1 inhibitor-treated C2C12 cells from panel C. Datapoints represent >10 technical replicates of one biological sample (*n*=1) and bars show the mean. (F) Western blot analysis of MyoG protein levels in C2C12 cells treated with DMSO and CARM1 inhibitor from panel D (*n*=1). Actin was used as a loading control.

Next, we explored the functional implications of the TAZ–CARM1 interaction within the context of myogenesis using the C2C12 myogenic differentiation model. Following the induction of myogenin (MyoG) protein expression, a well-characterized marker for the initiation of myogenic differentiation, we observed that although CARM1 remained enriched in the nucleus throughout differentiation (Fig. 3B), YAP/TAZ exhibited a progressive increase in their cytoplasmic localization in differentiating cells [cultured in differentiation medium (DM)] compared to the proliferative state [cultured in growth medium (GM)] (Fig. 3A; Fig. S2A–C). Next, we aimed to functionally assess the interaction using the well-characterized myogenin promoter-driven luciferase reporter gene assay in differentiating C2C12 cells. Consistent with our previous observations of TAZ acting as a repressor of the myogenic program, ectopic expression of FLAG–TAZ resulted in a decrease in myogenin reporter gene activation compared to that seen with transfection of the empty vector control (Fig. 3C). Expression of HA–CARM1 alone led to an increased fold change of the myogenin reporter, and when co-expressed with FLAG–TAZ, CARM1 counteracted TAZ repression, restoring reporter activation to normal levels (Fig. 3C). To further characterize the role of CARM1 in myogenesis, we treated C2C12 cells cultured for 48 h in DM with a selective inhibitor of CARM1 methyltransferase activity. We documented a decreased fraction of cells expressing MyoG in cells treated with the inhibitor compared with that in cells treated with the DMSO control (Fig. 3D,E). Western blot analysis indicated a decrease in MyoG protein levels in C2C12 cells treated with the CARM1 inhibitor compared to those in cells treated with the DMSO control (Fig. 3F).

### CARM1 methylates TAZ at Arg77 to promote Ser89 phosphorylation

**Fig. 4.**
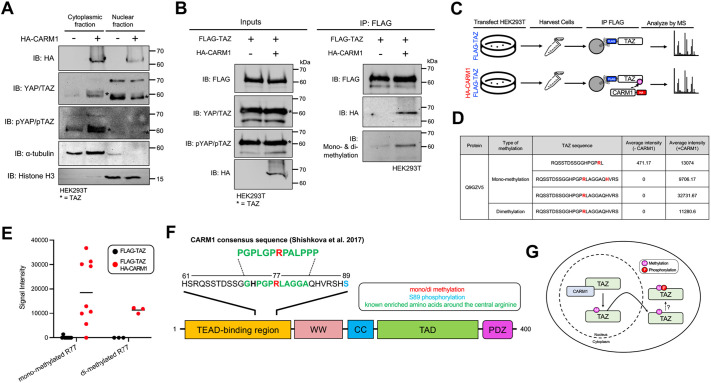
**Addition of CARM1 promotes TAZ Arg77 methylation and subsequent nucleocytoplasmic shuttling.** (A) HEK293T cells were transfected with the HA–CARM1 plasmid and subsequently harvested as cytoplasmic and nuclear fractions. Protein levels of HA–CARM1, YAP/TAZ, phosphorylated YAP at Ser127 (pYAP) and phosphorylated TAZ at Ser89 (pTAZ) were assessed by western blot analysis. α-tubulin and histone H3 were used as cytoplasmic and nuclear markers, respectively (*n*=1). Transfection with an empty vector (pcDNA3) served as a control. Bands representing endogenous TAZ are marked with asterisks. (B) FLAG–TAZ alone and combined with HA–CARM1 was ectopically expressed and immunoprecipitated using FLAG beads. Eluates were analyzed using western blot analysis for the contents of immunoprecipitated FLAG–TAZ using an antibody detecting protein methylation. Bands representing FLAG–TAZ are marked with asterisks. Blots represent two (A) and five (B) independent experiments. (C) Schematic illustrating the experimental design of mass spectrometry (MS)-based identification of methylated TAZ residues. (D) Table summarizing the intensity scores of TAZ peptide sequences containing methylated arginine residues in cells transfected with or without the HA–CARM1 plasmid. (E) Quantification of the signal intensity of TAZ sequences containing monomethylated and dimethylated arginine in cells transfected with or without the HA–CARM1 plasmid. Bars show the mean. (F) Illustration of the amino acid sequence of TAZ highlighting the methylated arginine residue (red) found within the TEAD-binding domain. (G) Schematic of the working model.

The balance of TAZ localization between the cytoplasm and nucleus is highly regulated by Ser89 phosphorylation by LATS kinases. In the canonical pathway, Ser89 phosphorylation leads to interaction with 14-3-3 proteins and robust cytoplasmic sequestration (Lei et al., 2008). To investigate whether CARM1 affects the localization of TAZ, we ectopically expressed HA–CARM1 and conducted subcellular fractionation of TAZ. The ectopic expression of HA–CARM1 resulted in a reduction of nuclear TAZ levels and an increase in cytoplasmic TAZ compared to the control, indicating a nucleocytoplasmic shift in TAZ localization upon ectopic expression of CARM1 (Fig. 4A). Additionally, TAZ phosphorylated at Ser89 was markedly increased in the cytoplasmic fraction of cells transfected with HA–CARM1, consistent with the observed increase in total cytoplasmic TAZ (Fig. 4A). We speculated that the methyltransferase activity of CARM1 might be coupled to the phosphorylation of TAZ at Ser89 and, therefore, TAZ localization. To investigate a potential mechanism underlying the differential phosphorylation of TAZ observed with ectopic expression of CARM1, we first assessed whether CARM1 directly methylates TAZ. We co-transfected HEK293T cells with FLAG–TAZ and HA–CARM1, followed by FLAG immunoprecipitation and western blotting using an antibody detecting protein methylation. TAZ methylation was enhanced in cells co-transfected with HA–CARM1, suggesting that TAZ is methylated by CARM1 (Fig. 4B). To further interrogate whether specific residues on TAZ are methylated by CARM1, we employed MS in conjunction with TAZ immunoprecipitation from cells expressing HA–CARM1 or control cells (Fig. 4C). Analysis of TAZ post-translational modification by tandem MS (using a timsTOF mass spectrometer) indicated monomethylation and dimethylation at Arg77 due to co-expression with CARM1 (Fig. 4D–G). Indeed, the amino acid sequence surrounding the central methylated arginine (Arg77) aligns with a previous report that demonstrates an enrichment of prolines, glycines, alanines and phenylalanines in the peptide surrounding the central methylated arginine (Shishkova et al., 2017). To confirm that the anti-TAZ band in the eluate sample corresponded specifically to FLAG–TAZ, rather than an auto-methylated CARM1 band co-precipitating with TAZ during the immunoprecipitation, we co-transfected HEK293T cells with HA–TAZ and HA–CARM1. This allowed us to assess the resolution of TAZ and CARM1 by SDS-PAGE, which successfully demonstrated clear separation between the two proteins (Fig. S3). In conclusion, these findings support the idea that TAZ is a substrate for CARM1, and that methylation enhances TAZ Ser89 phosphorylation leading to cytoplasmic sequestration.

## DISCUSSION

The Hippo signaling pathway has garnered considerable interest of late as a critical regulator of organ size control and potential modulator of stem cell regenerative potential (Heng et al., 2020). The canonical Hippo pathway culminates with the downstream regulation of transcriptional co-regulators YAP/TAZ, which serve to regulate Hippo-dependent gene expression and also integrate the Hippo pathway with the Wnt, TGF-β and Notch signaling pathways (Attisano and Wrana, 2013; Kim and Jho, 2014; Totaro et al., 2018). Without direct DNA-binding properties, TAZ cooperates extensively with sequence-specific DNA-binding proteins and other co-regulators to exert control over gene regulation. Thus, TAZ function is quite dependent on its cohort of interacting proteins. Using a nanobody-mediated AP approach coupled with LC–MS/MS, we report interactome datasets for TAZ in cardiomyocyte and skeletal myogenic cells. Among the identified interactome components in muscle cells, we further investigated an interaction with the methyltransferase CARM1. We demonstrate that CARM1 modulates TAZ repression of the myogenic differentiation machinery and directly methylates TAZ, leading to enhanced Ser89 phosphorylation and cytoplasmic sequestration. Due to the robust identification of previously well-characterized TAZ-interacting proteins, we postulate that the AP–LC–MS/MS approach we have utilized in these studies documents a generally applicable and highly robust strategy for protein interactome analysis.

Comparing our interactome findings with previous TAZ interactome studies from different cell types reveals a core interactome highlighted by TEAD transcription factors, LATS kinases, PDZ proteins, 14-3-3 chaperone proteins and SWI/SNF complex components (Kohli et al., 2014; Wang et al., 2014; Li et al., 2020). Several candidates identified in our study that merit future characterization in a myogenic context are the components of the chromatin remodeling NuRD complex. The NuRD complex decreases chromatin accessibility and transcriptional activity by coupling ATP-dependent chromatin remodeling with histone deacetylase activity (Xue et al., 1998). The repressor function of YAP/TAZ has, in some contexts, been shown to involve the recruitment of the NuRD complex through interactions between YAP/TAZ and MTA subunits (Kim et al., 2015). Interestingly, a NuRD–TLE–TBX20 repressor complex has also been discovered to function during embryonic heart development (Kaltenbrun et al., 2013). This possibility is supported in our studies as the NuRD subunits and TLE3 were identified in our TAZ interactome profile.

Extensive evidence on TAZ function and regulation has revealed the primary mechanism of TAZ repression due to Hippo pathway activation is by phosphorylation at Ser89 leading to TAZ sequestration and degradation in the cytoplasm (Lei et al., 2008). In this report, we propose CARM1-mediated methylation as a novel repressive regulatory mechanism of TAZ. This mechanism might contribute to the pro-myogenic function of CARM1 as nuclear TAZ function in myoblasts prevents myogenesis by targeting and repressing the myogenic transcription machinery (Tripathi et al., 2022). Several other studies have established CARM1 as a positive regulator of the myogenic program, including its functional interactions with the transcription factors MEF2 (Chen et al., 2002) and Pax7 (Kawabe et al., 2012; Chang et al., 2018b). CARM1 depletion by morpholino injection led to reduced *myogenin* expression in the anterior somites of developing zebrafish (Batut et al., 2011), consistent with our observations that CARM1 upregulates a myogenin promoter reporter gene. Our fractionation and methylation assay data reveal that, upon expression of ectopic CARM1, TAZ is methylated at Arg77 and adopts a Ser89 phosphorylation-enhanced cytoplasmic localization, suggesting the possibility that CARM1 represses TAZ during the onset of myogenesis to functionally counteract TAZ repression of the myogenic program. Indeed, monomethylation of the TAZ paralog YAP at Lys494 by the SET domain-containing lysine methyltransferase Set7 (also known as SETD7) has been shown to be necessary for retention of YAP in the cytoplasm, promoting its phosphorylation and subsequent degradation (Oudhoff et al., 2013). These observations in myoblasts could have important implications for the postnatal muscle regeneration program as activated adult muscle stem cells (satellite cells) go through a proliferative phase before committing to the differentiation program. It will be intriguing to assess whether Hippo or TAZ regulation plays a role in this context.

In this study, we identified TAZ-interacting proteins in skeletal muscle myoblasts and primary cardiomyocytes. Although we focused most of our attention on the downstream role of TAZ-interacting proteins in myogenesis, the role of interactome components in cardiomyocytes might also prove to be important for understanding cardiac gene regulation. The Hippo pathway plays a pivotal role in the development, disease and regeneration of the heart (Heallen et al., 2011, 2013; Leach et al., 2017; Chen et al., 2020). YAP is a well-known stimulator of cardiomyocyte proliferation, suggesting a possible approach to overcoming the limited potential for heart regeneration and repair following injury (Xin et al., 2013; Monroe et al., 2019). Cardiac-specific YAP deletion impedes embryonic heart development while, conversely, mice with cardiac-specific TAZ deletion are viable, suggesting that the roles of YAP and TAZ in heart differ (Xin et al., 2013). A conditional deletion of TAZ in zebrafish results in reduced cardiomyocyte proliferation and cardiac trabeculation (Lai et al., 2018). The only TEAD member appearing in the cardiomyocyte TAZ interactome was TEAD1, the most abundant TEAD family member with nonredundant roles in cardiac development (Chen et al., 1994; Liu et al., 2017). TEAD1 has been shown to enhance the reprogramming efficiency of cardiomyocytes from cardiac fibroblasts (Singh et al., 2021). Furthermore, TAZ and TEAD1 co-migrate to the nucleus in cardiomyocytes derived from hearts with desmin mutation (Hou et al., 2017). These observations indicate a potential association between TAZ and TEAD1 in the heart. Other interesting inclusions in the cardiomyocyte interactome are the cohesin (SMC1A) and condensin (SMC2 and SMC4) subunits, along with minichromosomal maintenance (MCM) proteins MCM2–MCM7. MCM proteins were shown recently to extrude cohesin-mediated DNA loops (Dequeker et al., 2022), suggesting that TAZ is also involved in regulating DNA looping in cardiomyocytes.

The affinity-capture method used here offers a number of advantages for AP–LC–MS/MS analysis. First, this approach can be applied to model systems that are difficult to transfect, such as primary cells (particularly cardiomyocytes). By using an easy-to-transfect cell line such as HEK293T, robust expression and purification of the bait protein is achieved, bypassing transfection challenges associated with expressing tagged proteins in the cells of interest. Unlike other proteomic interactome workflows, this method avoids ectopic expression of the bait protein in the cells of interest, which can lead to stoichiometry issues, alteration of the target cell proteome and non-physiological interactions due to ectopic overexpression in the target cells. The approach reported herein thus maintains the unaltered and native state of the endogenous proteome in the prey protein lysate (target cells). Lastly, the flexibility of this approach enables interactome screening across diverse biological systems using a consistent bait protein. This standardization simplifies comparisons of interactomes from different cell types, as the bait protein remains constant, minimizing variability. This is particularly beneficial for comparative studies, high-throughput screens and exploring how interactions vary across different cell types or experimental conditions. In terms of caveats, it should be noted that the bait protein is produced in HEK293T cells and might not be post-translationally modified in the same manner as the native protein in the target cells (prey lysate). This could lead to the identification of non-physiological interactions. However, production of the bait protein in HEK293T cells minimally maintains normal mammalian cell processing and basal modification of the protein, which one would consider an advantage over using bacterially expressed proteins (e.g. GST-tagged) as bait that would not be processed or modified in a similar manner. Obviously, there are pros and cons of many interactome methods, therefore necessitating careful further validation of interactions using co-precipitation and functional assays.

Overall, this study provides further evidence of TAZ as a modulator of myogenic transcription. Upon differentiation, our data implicate CARM1-mediated methylation and repression of TAZ nuclear function in order to facilitate de-repression of the myogenic differentiation machinery. In addition, based on the success of this approach to document the TAZ protein interactome described here and the widely available components of the nanotrap system and EYFP bait proteins, we contend that the AP–MS/MS approach we have utilized in these studies documents a generally applicable and highly robust strategy for protein interactome analysis.

## MATERIALS AND METHODS

### Cell line culture

C2C12 myoblasts and HEK293T cells were obtained from the American Type Culture Collection. Cells were cultured in growth medium (GM) consisting of high-glucose Dulbecco's modified Eagle's medium (DMEM, Gibco) and 10% fetal bovine serum (FBS, Thermo Fisher Scientific), supplemented with 1% penicillin/streptomycin (Invitrogen, Thermo Fisher Scientific). C2C12 myotube formation was induced by replacing GM with differentiation medium (DM), consisting of DMEM supplemented with 2% FBS and 1% penicillin/streptomycin. Cells were maintained in a humidified incubator at 5% CO_2_ and 37°C and replenished with fresh medium every 48 h.

### Primary cardiomyocyte isolation

Neonatal rat cardiomyocytes (NRCMs) were prepared from 1- to 3-day-old Sprague Dawley rats using the Neonatal Cardiomyocyte Isolation system (Worthington Biochemical, Lakewood, NJ, USA). Briefly, whole hearts (eight to 16) were dissociated with trypsin (Promega, Madison, WI, USA) and collagenase (Worthington Biochemical). The cells were re-suspended in DMEM F12 (Gibco) supplemented with 10% FBS, 1% penicillin/streptomycin and 50 mg/l gentamycin sulfate (Invitrogen). The isolated cells were plated for 60 min in a 37°C humidified incubator with 5% CO_2_ in air, allowing differential attachment of non-myocardial cells. Cardiomyocytes were counted using a hemocytometer, then transferred to gelatin-coated plates. The next day, the culture medium was removed and replaced with fresh medium. The following day, the medium was replaced with fresh serum-free media before subsequent experimentation.

### Transfections

For ectopic protein expression in HEK293T cells, the cells were transfected using polyethyleneimine (1 mg/ml, Sigma) at a polyethyleneimine:DNA ratio of three. C2C12 myoblasts were transfected with Lipofectamine 2000 (Life Technologies) at a Lipofectamine 2000:DNA ratio of three. All cells were re-fed with fresh culture medum at 16 h post transfection and harvested the next day.

### Antibodies and reagents

The following primary antibodies were used in this study: anti-YAP/TAZ (rabbit polyclonal, #D24E24), anti-phosphorylated TAZ (Ser89) (rabbit monoclonal, #59971), anti-HA (rabbit polyclonal, #C29F4), anti-Myc (mouse monoclonal, #9B11), anti-α-tubulin (rabbit polyclonal, #2144S), anti-c-myc (rabbit monoclonal, #5605), anti-CARM1 (rabbit monoclonal, #3379), anti-CARM1 (mouse monoclonal, #12495) and anti-histone H3 (rabbit polyclonal, #9715) from Cell Signaling Technology; anti-MyoG (mouse monoclonal, #F5D) and anti-β-actin (mouse monoclonal, #sc-47778) from Santa Cruz Biotechnology; anti-FLAG (mouse monoclonal, #F3165) from Sigma-Aldrich; anti-TLE3 (rabbit polyclonal, #22094-1-AP) from Proteintech; anti-GFP (rat monoclonal, #3H9) from ChromoTek; and anti-mono and dimethyl arginine (mouse monoclonal, ab412) from Abcam. All primary antibodies were used at a concentration of 1:1000. The CARM1 inhibitor (Sigma-Aldrich, #217531) was used at a concentration of 5 μM.

### Plasmids

The following plasmids were acquired from Addgene: Myc–TEAD1 (#33109) and Myc–TEAD4 (#24638) (deposited by Kunliang Guan; Zhao et al., 2008); FLAG–TAZ (#24809; deposited by Jeff Wrana; Varelas et al., 2008); HA–TAZ (#32839; deposited by Kunliang Guan; Lei et al., 2008); FLAG–MTA2 (#140964; deposited by Brian Hendrich; Burgold et al., 2019); pcDNA3 (#10792; deposited by William Sellers); HA–CARM1 (#81118; deposited by Sung Hee Baek; Shin et al., 2016); HOP-flash (#83467) and HIP-flash (HOP-flash mutant) (#83466) (deposited by Barry Gumbiner; Kim and Gumbiner, 2015); and myogenin core promoter (#134722; deposited by Michael Chin; Sun et al., 2001). Renilla (pRL-Renilla) plasmid was purchased from Promega. The TLE3 open reading frame was amplified by PCR using cDNA derived from C2C12 cells with BamHI- and Xho1-incorporated primers, and digested and inserted into pcDNA3 (Invitrogen).

### Cell harvesting

C2C12, rhabdomyosarcoma (RD), HEK293T and NRCM cells were harvested using NP-40 lysis buffer [150 mM NaCl, 1.0% (v/v) NP-40, 50 mM Tris (pH 8.0) and inhibitors] or, if destined for the gene reporter assay, with 1× reporter lysis buffer (Promega, #E4030). Cells were washed three times with ice-cold PBS before adding in lysis buffer. Lysates were collected and vortexed at 4°C for 15 min. Lysates were then centrifuged at 4°C and 12,000 ***g*** for 10 min, followed by collection of the supernatant containing the soluble protein.

### GFP-nanotrap sample preparation for MS

Bait protein (EYFP–TAZ and GFP) constructs were transfected in HEK293T cells and harvested the next day with NP-40 lysis buffer. 100 μg of extracted total protein was incubated with GFP-Trap magnetic agarose beads (ChromoTek, gtma-100) at 4°C on a rotator for 1 h. Beads were washed twice with 500 μl RIPA buffer at 4°C for 5 min, followed by another wash with 800 μl NP-40 lysis buffer at 4°C for 10 min. Prey proteins from C2C12 cells and NRCMs were harvested as described above. GFP-Trap beads, now with immobilized bait protein, were incubated with 1 mg of prey proteins at 4°C on a rotator overnight. Beads were washed three times with 1 ml of NP-40 lysis buffer, rotating at 4°C for 5 min, followed by a final wash with 1× PBS, rotating at 4°C for 5 min. MS analysis of the TAZ interactome was performed by the SPARC BioCentre (Molecular Analysis), The Hospital for Sick Children, Toronto, Canada.

### FLAG immunoprecipitation sample preparation for MS

HEK293T cells were cultured to 70% confluency and subsequently transfected with the FLAG–TAZ plasmid or the FLAG–TAZ and HA–CARM1 plasmids. Cells were harvested using lysis buffer and 1 mg of the resulting lysates was incubated on a rotator at 4°C overnight with anti-FLAG M2 magnetic beads (Sigma-Aldrich, #M8823) that had been washed three times with PBS. Lysates were removed the next day and beads were washed twice with 0.1% acetic acid on a rotator at 4°C for 3 min. Samples were then incubated with 50 mM NH_4_HCO_3_ and 4 mM dithiothreitol (DTT) for 30 min at 60°C, followed by a cooling period to room temperature. Iodoacetamide was added to the solution to a final concentration of 10 mM and the samples were incubated in the dark for 15 min, followed by further addition of DTT to a final concentration of 40 mM to inactivate residual iodoacetamide activity. The pH of the solution was measured to ensure that it was between 7.5 and 8.5. Digestion using MS-grade trypsin (Thermo Fisher Scientific, cat. #90057) was performed overnight at 37°C.

### LC–MS/MS analysis of TAZ methylation status

LC–MS/MS experiments were performed on a timsTOF Pro 2 (Bruker) mass spectrometer with a nanoElute 2 LC system (Bruker) and the HyStar software suite (Bruker). Separation was performed on a 10 cm×150 µm C18 column (PepSep Ten unlimited series, Bruker) with a particle size of 1.5 µm and pore size of 100 Å. A 5 mm×300 µm trap column was used (PepMap TM C18, Themo Fisher Scientific) with a particle size of 5 µm and pore size of 100 Å. Separation was performed using a two-column separation method using HyStar, including solvent preparation, column equilibration and sample-loading steps. The solvents used were water with 0.1% formic acid (mobile phase A), and acetonitrile with 0.1% formic acid (mobile phase B). The gradient started at 2% B to 35% B in 45 min, then to 95% B in 0.5 min for 1.68 min. The flow rate was 0.5 µl/min throughout the run. Data folders were processed using PaSER 2023 (Thermo Fisher Scientific) using the ‘DDA – ProluCID-GPU’ search engine and the UniProt human proteome database (accessed March 19th 2021). The MS analysis of TAZ methylation status was carried out by the YSciCore facility at York University, Toronto, Canada.

### Western blot analysis

Protein samples were denatured in 3× SDS loading buffer at 100°C for 10 min. Samples were then loaded onto 10% SDS-PAGE gels and ran at 100 V for approximately 1 h. The gel was transferred to a PVDF membrane (Millipore) in 1× transfer buffer and blocked in 5% blocking buffer for 1 h on an orbital shaker at room temperature. Primary antibodies were prepared by diluting the indicated antibody in 1% blocking buffer (1:1000). Membranes were probed with primary antibody solutions overnight at 4°C on a rocker. After brief washing with TBS supplemented with 0.1% Tween 20 (TBS-T), blots were incubated with corresponding HRP-conjugated secondary antibody in 1% blocking buffer (1:2000, Cell Signaling, #58802S) for 1 h at room temperature. After three washes with TBS-T, protein/antibody immune-complexes were detected by incubating blots in HRP substrate (Bio-Rad) and exposing them to an iBright CL1500 Imaging System (Thermo Fisher Scientific). All immunoblot images are documented in Fig. S4.

### Co-immunoprecipitation

For endogenous co-immunoprecipitation, Dynabeads Protein G (Invitrogen, #10003D) were washed three times with PBS before incubation with the anti-YAP/TAZ antibody (Cell Signaling Technology, #D24E4; diluted to 1:2000 with 1× PBS) or rabbit IgG (Cell Signaling Technology, #2729; 1:1000) on a rotator at 4°C overnight. Beads were washed three times with PBS to remove unbound antibody. Dynabeads were incubated with 1 mg of C2C12 myoblast extracts on a rotator at 4°C overnight. Beads were then washed three times with PBS to remove unspecific protein binding. Samples were eluted by heating with SDS loading buffer for 10 min at 95°C and analyzed by western blotting. For FLAG co-immunoprecipitations, anti-FLAG M2 magnetic beads (Sigma-Aldrich, #M8823) were washed three times with PBS before incubation with 500 μg of HEK293T cell extracts on a rotator at 4°C overnight. Beads were then washed three times with PBS to remove unspecific protein binding. Immunoprecipitates were eluted in 500 μg/ml FLAG 3× peptide solution (Sigma-Aldrich, #4799) on a rotator at room temperature for 30 min and analyzed by western blotting.

### Cell fractionation

C2C12 cells were grown in GM until 90% confluency and then in DM for 4 days. Myotubes were isolated by incubating the cells with 0.1% ice-cold trypsin for 30 s and removing the detached myotubes. The remaining reserve cells were removed using a cell scraper. Nuclear and cytoplasmic fractions of the myotube and reserve cell fractions were harvested using the NE-PER kit (Thermo Fisher Scientific, #78833) according to the manufacturer's instructions.

### Immunofluorescence

C2C12 cells were seeded on glass-bottomed dishes (MatTek) and washed with ice-cold PBS three times, followed by fixation with 4% paraformaldehyde at room temperature for 10 min. Cells were washed with PBS and permeabilized with ice-cold 90% methanol on ice for 5 min. Cells were washed three times with PBS and incubated with immunofluorescence (IF) blocking buffer (5% FBS in PBS) for 1 h 30 min at room temperature, then incubated with the indicated primary antibodies in IF blocking buffer at 4°C overnight. Cells were washed the next day to remove unbound antibodies and incubated with Alexa Fluor-conjugated secondary antibodies (Life Technologies) in IF blocking buffer for 1 h 30 min. Cells were washed three times with PBS and counterstained with Hoechst 33342 for 10 min. Cells were subjected to imaging with a Zeiss Observer Z1 confocal fluorescence microscope equipped with a Yokogawa CSU-XI spinning disk. Images were recorded using a AxioCam MRm camera (Zeiss) and processed by Zen 2.5 (blue edition) software (Zeiss).

### Luciferase reporter gene assays

Transcriptional reporter assays were performed using luciferase reporter plasmids along with expression constructs (indicated in the figure legends) and a Renilla plasmid (Promega, pRL-Renilla) as an internal transfection control. Cells were washed with ice-cold PBS and harvested using 1× reporter lysis buffer (Promega, #E4030). Enzymatic activity was measured in each sample on a Lumat LB luminometer (Berthold Technologies) using luciferase assay substrate (Promega, #E1501) or Renilla assay substrate (Promega, #E2820). Luciferase activity values were normalized to Renilla activity values in the same cell extracts and expressed as fold activation to the control.

### Statistical analysis

Statistical analysis was conducted on PRISM from GraphPad 10.0. Using this software, an unpaired one-tailed *t*-test was used to test for statistical significance. Adjusted *P*-values (*P≤0.05, **P≤0.01, ***P≤0.001, ****P<0.0001) are indicated for significance compared to the relevant control condition.

## Supplementary Material



10.1242/joces.263527_sup1 Supplementary information
