# Supplementary material for: TAZ interactome analysis using nanotrap-based affinity purification–mass spectrometry
Source: J Cell Sci. 2025 Feb 24;138(4):jcs263527. doi: 10.1242/jcs.263527 (PMC11928053; doi:10.1242/jcs.263527)
Supplement: Supplementary information [file joces-138-263527-s1.pdf]

C2C12 (152)

ABCD3	CCDC47	DYNC1H1	GMPS	IGF2BP1	MRE11	PKM	PTRH1	SEC23A	TCERG1	ZCCHC8
ACAT1	CDK1	EEF2	GNL1	ILF2	MRPL15	PKN2	RO60	SEC23B	TEAD1	ZFR
ACOT7	CHD1	EIF3H	GNL2	KHSRP	MRPS9	PNKP	RAB5A	SEC24C	TFRC	
ACSL3	CHERP	ELOC	GTF2I	KPNA1	MYCBP2	POLB	RAB8A	SEC31A	TIAL1	
ACTR2	CHTOP	EMC1	GTF3C1	LATS1	NCKAP1	POLR1C	RAC1	SF3B3	TIMM50	
ADSS2	CNOT9	ENO1	GTF3C3	LDHA	NDUFA10	POLR2L	RAD50	SF3B4	TJP1	
AHSA1	COPS2	ERGIC1	GTPBP1	LDHB	NME1	PPP2CA	RAN	SLIRP	TUBA1C	
AP3M1	COPZ1	ETF1	HCFC1	LIG3	NPEPPS	PPP2R1A	RAP1B	SMARCAD1	TUBB2B	
API5	CPSF3	EXOSC10	HDAC1	LLGL1	OSBPL8	PPP2R2A	RBBP4	SNRNP70	TUBB6	
ARF1	CSE1L	EXOSC4	HK2	LSM12	PAF1	PRPF38A	RBM10	SNRPA1	TUBG1	
ARL8B	CTR9	FERMT2	HSPA1B	LTV1	PARP2	PRPF40A	RBM25	SRM	UQCRQ	
ARPC2	CYC1	FUBP3	HSPA4L	LUC7L2	PDHA1	PRPS1L3	RBM27	SRPRB	WDR6	
BCLAF1	DDX41	GANAB	HUWE1	MFAP1B	PFAS	PSMC1	RPA3	SRRT	WDR82	
C1QBP	DKC1	GAPVD1	IARS1	MPC2	PGD	PSMD11	RUVBL1	SRSF9	XRCC1	
CALM1	DNAJA3	GLG1	IDH3B	MPDZ	PIGK	PSMD3	SEC22B	ST13	YWHAQ	

PCM (119)

ABCE1	ATP6V1B2	CPSF1	EIF3C	FTSJ3	IGF2BP3	MCM5	NOP58	PHGDH	RBFOX2	SF3B2	STRAP
ABCF2	BCAS2	CSDE1	EIF3D	GNB2	IK	MCM7	NSUN2	PLRG1	RBM15	SFXN1	SUPT16H
ADSS	CAPN1	CSNK2A2	EIF3E	GRWD1	IMPDH1	MRPL16	OTUB1	POLDIP3	RBM25L1	SMARCA5	TARDBP
AFDN	CBX3	CTTN	EIF3F	GYS1	IPO5	MRPL40	P4HA1	PRKAR2B	RBMX	SMARCD1	TIMM44
AGL	CCT6A	DDX47	EIF4A3	HDAC1L	IQGAP1	MRPS7	PARP1	PRMT1	RCC2	SMC2	TRIM28
ALDH18A1	CHD4	DHX15	FARSA	HNRNPLL	LARS	MTCH2	PARP12	PRPF19	RNPS1	SMC4	TUBB4A
ALYREF	CHTOPL1	DIS3	FBN1	HNRNPR	LRRC47	MTHFD1L	PCNA	PSPC1	RPL36A	SMU1	U2AF1
ANKRD17	CKB	DNAJA1	FIP1L1	HNRNPUL2	MATR3	MTREX	PFKL	PURA	RPL39	SRP14	USP10
AP3B1	CNOT1	EDC4	FLNB	IARS	MCM3	NARS	PFKM	RAB7A	RPLP1	SRRM2	ZFHX3
ASCC3	COPG1	EIF3A	FMR1	IGF2BP2	MCM4	NOP2	PGAM5	RBBP7	SCFD1	SRSF7	

BOTH (97)

ACLY	ASNS	CTPS1	EFTUD2	HSP90AA1	MTA2	PRMT5	PSMD2	SCRIB	TAGLN2	USP7
ADSL	CAD	CYFIP1	EIF4G2	HSPA1L	NAA15	PRPF4B	PTGES3	SEC23IP	TARS1	XPO1
AHCY	CAND1	DDX39B	FASN	HSPH1	PAICS	PRPF6	PUM1	SF3B1	TJP2	YWHAB
AHCYL1	CARM1	DDX50	FBL	IMPDH2	PCMT1	PRPF8	PYGL	SMARCA4	TLE3	YWHAE
AMOT	CCAR2	DHX30	GARS1	KPNB1	PDCD6IP	PSMC2	RAB1A	SMARCC1	U2AF2	YWHAG
AP1M1	CNN3	DHX38	GART	LARP7	PLS3	PSMC3	RAB5C	SMC1A	U2SURP	YWHAH
AP2B1	COPB1	DHX9	GFM1	MCM2	POLR2B	PSMC4	RBM17	SMC3	UBE2M	YWHAZ
ARCN1	CSNK1A1	DNM2	GSPT1	MCM6	PRDX6	PSMC5	RHOA	SNRNP200	UBE2O	
ARID1A	CSNK2B	DOCK7	HPRT1	MRPS22	PRKDC	PSMC6	RUVBL2	SSRP1	UBR4	

Fig. S1. Complete list of proteins enriched by TAZ interactome analysis.

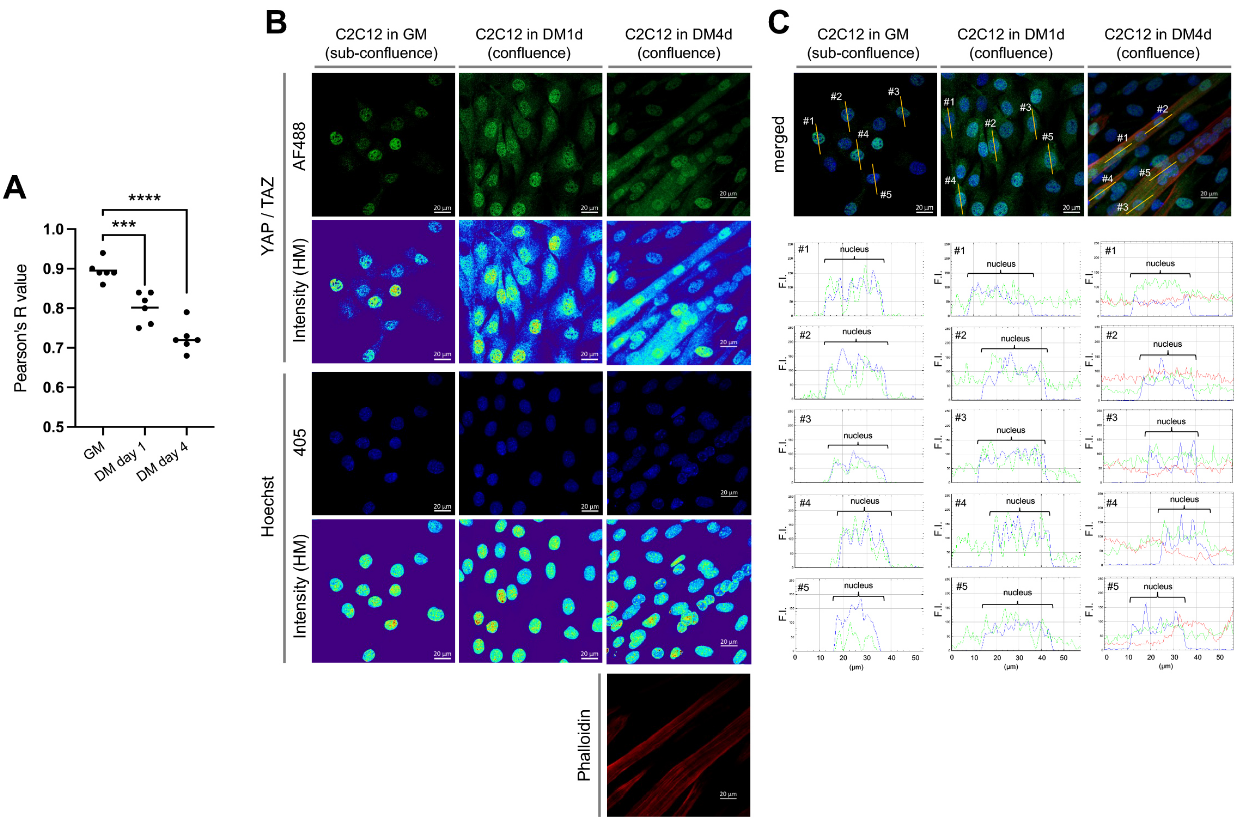

Fig. S2. C2C12 cells were seeded onto glass-bottom dishes at equal density. Cells were fixed at the indicated time points and conditions, then subjected to immunofluorescence (IF) analysis for YAP/TAZ localization. Nuclei were stained with Hoechst 33342, and F-actin accumulation was visualized using Phalloidin (DM 4d) to mark myotubes. Images were obtained with a Zeiss confocal spinning disc microscope and rendered in Zen Blue. **(A)** The degree of co-localization between the nuclear signal (Hoechst, blue) and YAP/TAZ signal (green) was analyzed using ImageJ (version 2.14.0/1.54f, Analyze > Colocalization > Coloc 2), with Pearson's R values calculated and graphed (n=6). **(B)** Fluorescence images of YAP/TAZ and Hoechst were converted to a heat map scheme based on signal intensity using Zen Blue (Zeiss). Micrographs represent 6 replicates. **(C)** YAP/TAZ sub-cellular localization in panel B was assessed by scanning fluorescence signal intensities across nuclei along a designated line (yellow). Signal intensity profiles for YAP/TAZ (green), Hoechst (blue), and Phalloidin (red) (DM4d only) were generated using ImageJ (Plugins > RGB Profiler). Nuclear regions were identified by Hoechst (blue) signal (n=5).

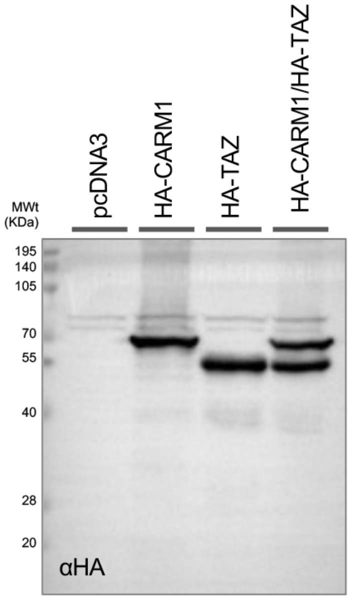

Fig. S3. Western blot analysis of HEK293T cells transfected with HA-TAZ and HA-CARM1.

Journal of Cell Science • Supplementary information

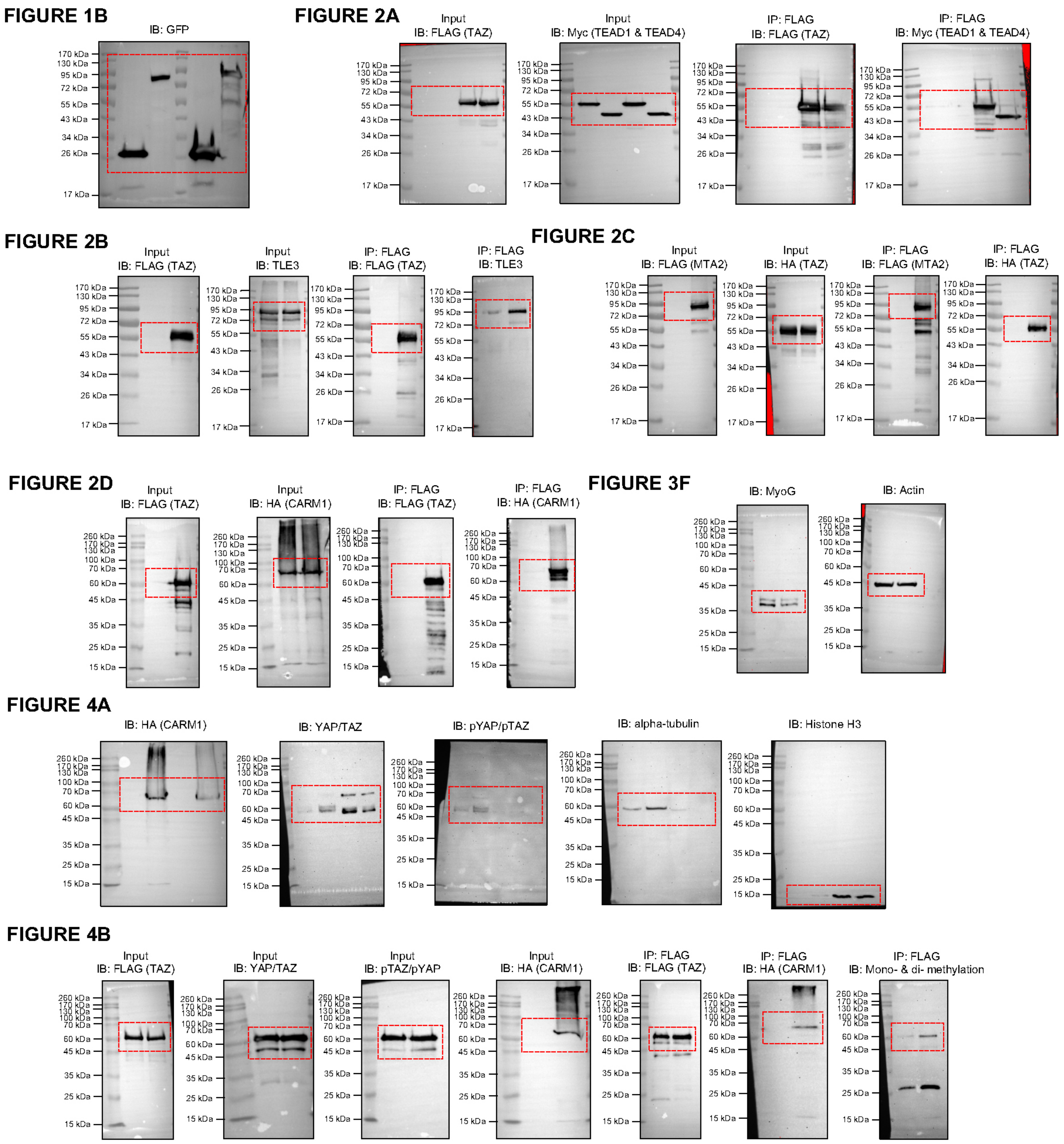

Fig. S4. Blot transparency for data shown in Figs. 1, 3, and 4. Red box indicates the region that was presented in corresponding figure.

Journal of Cell Science • Supplementary information

Table S1. Top 40 Ingenuity canonical pathways enriched in the C2C12 TAZ interactome dataset.

#	Ingenuity Canonical Pathway – C2C12	-log(p-value)	Proteins
1	Inhibition of ARE-Mediated mRNA Degradation Pathway	16.2	Cnot9, Exosc10, Exosc4, Ppp2ca, Ppp2r1a, Ppp2r2a, Psmc1, Psmc2, Psmc3, Psmc4, Psmc5, Psmc6, Psmd11, Psmd2, Psmd3, Xrn1, Ywhab, Ywhae, Ywhag, Ywhah, Ywhaq, Ywhaz
2	HIPPO signaling	15.5	Amot, Lats1, Llgl1, Ppp2ca, Ppp2r1a, Ppp2r2a, Scrib, Stk4, Tead1, Tead3, Tjp2, Ywhab, Ywhae, Ywhag, Ywhah, Ywhaq, Ywhaz
3	Purine Nucleotides De Novo Biosynthesis II	10.9	Adsl, Adss2, Gart, Gmps, Impdh2, Paics, Pfas
4	BAG2 Signaling Pathway	9.31	Hsp90aa1, Hspa1b, Hspa1l, Psmc1, Psmc2, Psmc3, Psmc4, Psmc5, Psmc6, Psmd11, Psmd2, Psmd3
5	Protein Ubiquitination Pathway	8.79	Dnajb1, Eloc, Hsp90aa1, Hspa1l, Hspa4l, Hsph1, Psmc1, Psmc2, Psmc3, Psmc4, Psmc5, Psmc6, Psmd11, Psmd2, Psmd3, Ube2m, Ube2o, Usp7, Usp9x
6	Cell Cycle: G2/M DNA Damage Checkpoint Regulation	8.04	Cdk1, Ep300, Prkdc, Ywhab, Ywhae, Ywhag, Ywhah, Ywhaq, Ywhaz
7	Remodeling of Epithelial Adherens Junctions	7.98	Actr2, Arpc2, Dnm2, Nme1, Rab5a, Rab5c, Tuba1c, Tubb2b, Tubb6, Tubg1
8	Huntington's Disease Signaling	7.84	Clta, Dnajb1, Dnm2, Ep300, Hdac1, Hspa1b, Hspa1l, Polr2b, Polr2l, Psmc1, Psmc2, Psmc3, Psmc4, Psmc5, Psmc6, Psmd11, Psmd2, Psmd3
9	FAT10 Signaling Pathway	7.59	Psmc1, Psmc2, Psmc3, Psmc4, Psmc5, Psmc6, Psmd11, Psmd2, Psmd3
10	RAN Signaling	7.44	Cse1l, Kpna1, Kpnb1, Ran, Ranbp1, Xpo1
11	14-3-3-mediated Signaling	7.23	Pdcd6ip, Rap1b, Tuba1c, Tubb2b, Tubb6, Tubg1, Ywhab, Ywhae, Ywhag, Ywhah, Ywhaq, Ywhaz
12	Polyamine Regulation in Colon Cancer	7.19	Psmc1, Psmc2, Psmc3, Psmc4, Psmc5, Psmc6, Psmd11, Psmd2, Psmd3
13	Spliceosomal Cycle	6.86	Ddx39b, Dhx38, Eftud2, Sf3b1, Sf3b3, Sf3b4, Snrnp200, U2af2
14	Hereditary Breast Cancer Signaling	6.7	Arid1a, Cdk1, Ep300, Hdac1, Mre11, Polr2b, Polr2l, Rad50, Rap1b, Smarca4, Smarcc1, Tubg1
15	DNA Double-Strand Break Repair by Non-Homologous End Joining	6.3	Lig3, Mre11, Prkdc, Rad50, Xrcc1
16	p70S6K Signaling	6.13	Eef2, Ppp2ca, Ppp2r1a, Ppp2r2a, Rap1b, Ywhab, Ywhae, Ywhag, Ywhah, Ywhaq, Ywhaz
17	ATM Signaling	5.5	Cdk1, Mre11, Ppp2ca, Ppp2r1a, Ppp2r2a, Rad50, Smc1a, Smc3, Usp7
18	Clathrin-mediated Endocytosis Signaling	5.28	Actr2, Ap1m1, Ap3m1, Arpc2, Clta, Csnk2b, Dnm2, Rab5a, Rab5c, Rac1, Tfrc, Usp9x
19	PI3K/AKT Signaling	5.15	Elp1, Hsp90aa1, Ppp2ca, Ppp2r1a, Ppp2r2a, Rap1b, Ywhab, Ywhae, Ywhag, Ywhah, Ywhaq, Ywhaz
20	BER pathway	4.99	Lig3, Pnkp, Polb, Xrcc1
21	AMPK Signaling	4.98	Arid1a, Eef2, Ep300, Fasn, Pfkl, Pfkm, Ppp2ca, Ppp2r1a, Ppp2r2a, Rab1a, Rab8a, Smarca4, Smarcc1
22	Glycolysis I	4.84	Aldoa, Eno1, Pfkl, Pfkm, Pkm
23	Mitotic Roles of Polo-Like Kinase	4.78	Cdk1, Hsp90aa1, Ppp2ca, Ppp2r1a, Ppp2r2a, Smc1a, Smc3
24	Coronavirus Replication Pathway	4.75	Arcn1, Copb1, Copz1, Tuba1c, Tubb2b, Tubb6
25	ERK5 Signaling	4.53	Rap1b, Ywhab, Ywhae, Ywhag, Ywhah, Ywhaq, Ywhaz
26	NER (Nucleotide Excision Repair, Enhanced Pathway)	4.39	Cops2, Ep300, Lig3, Polr2B, Polr2l, RpaA3, Usp7, Xrcc1
27	IGF-1 Signaling	4.36	Csnk2b, Rap1b, Ywhab, Ywhae, Ywhag, Ywhah, Ywhaq, Ywhaz
28	Telomerase Signaling	4.27	Dkc1, Hdac1, Hsp90aa1, Ppp2ca, Ppp2r1a, Ppp2r2a, Ptges3, Rap1b
29	HIF1α Signaling	4.24	Eloc, Ep300, Hsp90aa1, Hspa1b, Hspa1l, Ldha, Ldhb, Pkm, Rac1, Ran, Rap1b
30	Role of CHK Proteins in Cell Cycle Checkpoint Control	4.15	Cdk1, Mre11, Ppp2ca, Ppp2r1a, Ppp2r2a, Rad50
31	Tight Junction Signaling	4.12	Cpsf3, Llgl1, Mpdz, Ppp2ca, Ppp2r1a, Ppp2r2a, Rac1, Rhoa, Tjp1, Tjp2
32	Protein Kinase A Signaling	3.82	ACP1, Calm1, Ptpn23, Pygl, Pygm, Rap1b, Rhoa, Timm50, Ttn, Ywhab, Ywhae, Ywhag, Ywhah, Ywhaq, Ywhaz
33	Epithelial Adherens Junction Signaling	3.81	Actr2, Arpc2, Rac1, Rap1b, Rhoa, Tuba1c, Tubb2b, Tubb6, Tubg1

Journal of Cell Science • Supplementary information

34	Phagosome Maturation	3.79	Dync1h1, Prdx6, Rab5a, Rab5c, Rac1, Tuba1c, Tubb2b, Tubb6, Tubg1
35	Virus Entry via Endocytic Pathways	3.52	Ap1m1, Ap3m1, Clta, Dnm2, Rac1, Rap1b, Tfrc
36	Wnt/β-catenin Signaling	3.52	Csnk1a1, Csnk2b, Ep300, Hdac1, Ppp2ca, Ppp2r1a, Ppp2r2a, Ruvbl2, Tle3
37	Caveolar-mediated Endocytosis Signaling	3.49	Arcn1, Copb1, Copz1, Dnm2, Rab5a, Rab5c
38	ERK/MAPK Signaling	3.47	Ppp2ca, Ppp2r1a, Ppp2r2a, Rac1, Rap1b, Ywhab, Ywhag, Ywhah, Ywhaq, Ywhaz
39	5-aminoimidazole Ribonucleotide Biosynthesis I	3.35	Gart, Pfas
40	Inosine-5'-phosphate Biosynthesis II	3.35	Adsl, Paics

Table S2. Top 40 Ingenuity canonical pathways enriched in the NRCM TAZ interactome dataset.

#	Ingenuity Canonical Pathway	-log(p-value)	Proteins
1	Spliceosomal Cycle	14	Bcas2, Ddx39b, Dhx15, Dhx38, Eftud2, Eif4a3, Plrg1, Prpf19, Sf3b1, Sf3b2, Snrnp200, U2af2
2	Inhibition of ARE-Mediated mRNA Degradation Pathway	10.9	Cnot1, Dis3, Edc4, Prkar2b, Psmc2, Psmc3, Psmc4, Psmc5, Psmc6, Psmd2, Ywhab, Ywhae, Ywhag, Ywhah, Ywhaz
3	Purine Nucleotides De Novo Biosynthesis II	9.75	Adsl, Adss2, Gart, Impdh1, Impdh2, Paics
4	Protein Ubiquitination Pathway	6.85	Dnaja1, Hsp90aa1, Hspa1l, Hsph1, Psmc2, Psmc3, Psmc4, Psmc5, Psmc6, Psmd2, Ube2m, Ube2o, Usp10, Usp7
5	Cell Cycle Control of Chromosomal Replication	6.27	Mcm2, Mcm3, Mcm4, Mcm5, Mcm6, Mcm7, Pcna
6	BAG2 Signaling Pathway	6.16	Hsp90aa1, Hspa1l, Psmc2, Psmc3, Psmc4, Psmc5, Psmc6, Psmd2
7	HIPPO signaling	6.12	Amot, Scrib, Tjp2, Ywhab, Ywhae, Ywhag, Ywhah, Ywhaz
8	tRNA Charging	5.99	Farsa, Gars1, Iars1, Lars1, Nars1, Tars1
9	AMPK Signaling	5.79	Arid1a, Fasn, Gnb2, Gys1, Pfkl, Pfkm, Prkar2b, Rab1a, Rab7a, Smarca4, Smarcc1, Smarcd1
10	RAR Activation	5.74	Arid1a, Carm1, Csnk2a2, Csnk2b, Parp1, Prkar2b, Prmt1, Psmc5, Smarca4, Smarcc1, Smarcd1
11	IGF-1 Signaling	5.45	Csnk2a2, Csnk2b, Prkar2b, Ywhab, Ywhae, Ywhag, Ywhah, Ywhaz
12	Cell Cycle: G2/M DNA Damage Checkpoint Regulation	5.34	Prkdc, Ywhab , Ywhae, Ywhag, Ywhah, Ywhaz
13	Huntington's Disease Signaling	5.13	Capn1, Dnm2, Gnb2, Hdac1l, Hspa1l, Polr2b, Psmc2, Psmc3, Psmc4, Psmc5, Psmc6, Psmd2
14	FAT10 Signaling Pathway	5.05	Psmc2, Psmc3, Psmc4, Psmc5, Psmc6, Psmd2
15	Caveolar-mediated Endocytosis Signaling	4.31	Arcn1, Copb1, Copg1, Dnm2, Flnb, Rab5c
16	Methionine Degradation I (to Homocysteine)	4.29	Ahcy, Ahcyl1, Prmt1, Prmt5
17	Cysteine Biosynthesis III (mammalia)	4.15	Ahcy, Ahcyl1, Prmt1, Prmt5
18	Epithelial Adherens Junction Signaling	4.15	Afdn, Iqgap1, Rhoa, Ywhab, Ywhae, Ywhag, Ywhah, Ywhaz
19	Amyloid Processing	4.1	Capn1, Csnk1a1, Csnk2a2, Csnk2b, Prkar2b
20	EIF2 Signaling	3.81	Eif3a, Eif3c, Eif3d, Eif3e, Eif3f, Eif4a3, Eif4g2, Rpl39, Rplp1
21	Inosine-5'-phosphate Biosynthesis II	3.65	Adsl, Paics
22	ATM Signaling	3.64	Cbx3, Smc1a, Smc2, Smc3, Trim28, Usp7
23	Clathrin-mediated Endocytosis Signaling	3.53	Ap1m1, Ap2b1, Csnk2a2, Csnk2b, Cttn, Dnm2, Rab5c, Rab7a
24	Superpathway of Methionine Degradation	3.45	Ahcy, Ahcyl1, Prmt1, Prmt5
25	RAN Signaling	3.4	Ipo5, Kpnb1, Xpo1
26	DNA Methylation and Transcriptional Repression Signaling	3.36	Chd4, Hdac1l, Mta2, Rbbp7
27	ERK5 Signaling	3.33	Ywhab, Ywhae, Ywhag, Ywhah, Ywhaz
28	mTOR Signaling	3.26	Eif3a, Eif3c, Eif3d, Eif3e, Eif3f, Eif4a3, Eif4g2, Rhoa
29	Tetrahydrofolate Salvage from 5, 10-methenyltetrahydrofolate	3.13	Gart, Mthfd1l
30	14-3-3-mediated Signaling	3.07	Pdcd6ip, Ywhab, Ywhae, Ywhag, Ywhah, Ywhaz
31	Protein Kinase A Signaling	3.03	Flnb, Gnb2, Gys1, Prkar2b, Pygl, Rhoa, Ywhab, Ywhae, Ywhag, Ywhah, Ywhaz
32	Regulation of eIF4 and p70S6K Signaling	3.01	Eif3a, Eif3c, Eif3d, Eif3e, Eif3f, Eif4a3, Eif4g2
33	CSDE1 Signaling Pathway	2.85	Csde1, Cttn, Edc4, Pum1
34	Hereditary Breast Cancer Signaling	2.82	Arid1a, Hdac1l, Polr2b, Smarca4, Smarcc1, Smarcd1
35	PI3K/AKT Signaling	2.74	Gys1, Hsp90aa1, Ywhab, Ywhae, Ywhag, Ywhah, Ywhaz
36	Mitotic Roles of Polo-Like Kinase	2.58	Capn1, Hsp90aa1, Smc1a, Smc3
37	Remodeling of Epithelial Adherens Junctions	2.54	Dnm2, Iqgap1, Rab5c, Rab7a
38	Pyrimidine Ribonucleotides De Novo Biosynthesis	2.36	Cad, Ctps1, Dhx9
39	Glycogen Degradation II	2.33	Agl, Pygl
40	Role of BRCA1 in DNA Damage Response	2.28	Arid1a, Smarca4, Smarcc1, Smarcd1

Table S3. Top 20 KEGG pathways enriched in the C2C12 TAZ interactome dataset.

#	KEGG pathway	-log (FDR)	Proteins
1	Spliceosome	14.90	Prpf6, U2af2, Hspa1l, Prpf8, Snrpf, Eftud2, Sf3b1, Srsf9, Rbm17, Sf3b3, Dhx38, Rbm25, Snrnp70, Prpf40a, Sf3b4, Prpf38a, Cherp, U2surp, Snrnp200, Ppih, Snrpa1, Hspa1b, Ddx39b
2	Viral carcinogenesis	7.30	Scrib, Rhoa, Ywhab, Ywhah, Cdk1, Psmc1, Ywhaz, Polb, Pkm, Ywhag, Ep300, Ywhae, Rac1, Ubr4, Hdac1, Ywhaq, Ranbp1, Usp7
3	Hippo signaling pathway	7.06	Scrib, Ppp2r1a, Ywhab, Ywhah, Ppp2ca, Ywhaz, Ywhag, Tead1, Llgl1, Ywhae, Ppp2r2a, Ywhaq, Amot, Tead3, Lats1
4	Cell cycle	5.77	Ywhab, Ywhah, Cdk1, Ywhaz, Prkdc, Smc3, Smc1a, Ywhag, Mcm2, Ep300, Ywhae, Hdac1, Ywhaq
5	Proteasome	5.74	Psmd2, Psmd3, Psmd11, Psmc5, Psmc1, Psmc6, Psmc2, Psmc4, Psmc3
6	Tight junction	5.74	Actr2, Scrib, Rab8a, Ppp2r1a, Rhoa, Ppp2ca, Tuba1c, Llgl1, Rac1, Ppp2r2a, Tjp2, Tjp1, Mpdz, Amot
7	Huntington disease	5.74	Tubb6, Psmd2, Psmd3, Psmd11, Ap2b1, Psmc5, Psmc1, Psmc6, Cyc1, Ndufa10, Psmc2, Polr2b, Psmc4, Polr2l, Tuba1c, Ep300, Psmc3, Tubb2b, Hdac1
8	Prion disease	5.74	Tubb6, Psmd2, Hspa1l, Psmd3, Psmd11, Psmc5, Psmc1, Psmc6, Cyc1, Csnk2b, Ndufa10, Psmc2, Psmc4, Tuba1c, Psmc3, Tubb2b, Rac1, Hspa1b
9	Oocyte meiosis	4.59	Ppp2r1a, Ywhab, Ywhah, Cdk1, Ppp2ca, Ywhaz, Smc3, Smc1a, Ywhag, Ywhae, Ywhaq
10	Ribosome biogenesis in eukaryotes	4.28	Csnk2b, Nat10, Gnl2, Ran, Dkc1, Xrn1, Fbl, Mdn1, Xpo1
11	Amyotrophic lateral sclerosis	4.14	Tubb6, Rab8a, Psmd2, Psmd3, Psmd11, Rab5a, Psmc5, Psmc1, Psmc6, Cyc1, Ndufa10, Psmc2, Psmc4, Tuba1c, Psmc3, Tubb2b, Rac1, Rab1
12	Purine metabolism	4.11	Adss, Pfas, Adsl, Gart, Hprt, Gmps, Paics, Pkm, Impdh2, Nme1, Prps1l3
13	Parkinson disease	3.85	Tubb6, Psmd2, Psmd3, Psmd11, Psmc5, Psmc1, Psmc6, Cyc1, Ndufa10, Psmc2, Psmc4, Tuba1c, Psmc3, Tubb2b
14	mRNA surveillance pathway	3.70	Ppp2r1a, Wdr82, Ppp2ca, Pnn, Etf1, Cpsf3, Gspt1, Ppp2r2a, Ddx39b
15	Glucagon signaling pathway	3.64	Pfkl, Ldhb, Pdha1, Pkm, Pygm, Pfkm, Ep300, Pygl, Ldha
16	HIF-1 signaling pathway	3.29	Pfkl, Tfrc, Ldhb, Pdha1, Pfkm, Ep300, Aldoa, Ldha, Tceb1
17	Spinocerebellar ataxia	3.29	Psmd2, Psmd3, Psmd11, Psmc5, Psmc1, Psmc6, Pum1, Psmc2, Psmc4, Psmc3
18	Glycolysis / Gluconeogenesis	3.17	Pfkl, Ldhb, Pdha1, Pkm, Pfkm, Aldoa, Ldha
19	Endocytosis	3.17	Rab8a, Arpc2, Hspa1l, Rhoa, Rab5a, Ap2b1, Tfrc, Ehd1, Vps35, Arf1, Pdcd6ip, Dnm2, Hspa1b
20	Epstein-Barr virus infection	3.17	Psmd2, Psmd3, Psmd11, Psmc5, Psmc1, Psmc6, Psmc2, Psmc4, Psmc3, Rac1, Hdac1, Usp7

Table S4. Top KEGG pathways enriched in the NRCM TAZ interactome dataset.

#	KEGG pathway	-log(FDR)	Proteins
1	Spliceosome	15.27	Prpf6, U2af2, Bcas2, Hspa1l, U2af1, Prpf8, Eftud2, Sf3b2, Alyref, Eif4a3, Sf3b1, Dhx15, Rbm17, Dhx38, Srsf7, U2surp, Snrnp200, Rbmx, Plrg1, Ddx39b, Prpf19
2	RNA transport	9.19	Kpnb1, Eif3e, Prmt5, Eif3a, Alyref, Eif4a3, Cyfip1, Eif3c, Eif3f, Strap, Tardbp, Fmr1, Eif3d, Xpo1, Eif4g2, Rnps1, Ddx39b
3	Cell cycle	9.19	Mcm7, Ywhab, Ywhah, Ywhaz, Prkdc, Mcm4, Smc3, Mcm6, Pcna, Smc1a, Ywhag, Mcm3, Mcm2, Ywhae, Mcm5
4	DNA replication	4.94	Mcm7, Mcm4, Mcm6, Pcna, Mcm3, Mcm2, Mcm5
4	Aminoacyl-tRNA biosynthesis	3.30	Gars, Farsa, Tars, Nars, Lars, Iars
5	mRNA surveillance pathway	3.30	Alyref, Eif4a3, Cpsf1, Gspt1, Tardbp, Fip1l1, Rnps1, Ddx39b
6	Proteasome	3.30	Psmd2, Psmc5, Psmc6, Psmc2, Psmc4, Psmc3
7	Viral carcinogenesis	2.92	Scrib, Rhoa, Ywhab, Ywhah, Ywhaz, Ywhag, Chd4, Ywhae, Ubr4, Usp7
8	RNA degradation	2.20	Pfkl, Skiv2l2, Edc4, Dis3, Pfkm, Cnot1
9	Oocyte meiosis	2.20	Ywhab, Ywhah, Ywhaz, Smc3, Smc1a, Ywhag, Ywhae
10	Hippo signaling pathway	2.20	Scrib, Ywhab, Ywhah, Ywhaz, Ywhag, Ywhae, Amot,
11	Amyotrophic lateral sclerosis	2.08	Psmd2, Psmc5, Psmc6, Alyref, Psmc2, Psmc4, Srsf7, Psmc3, Tubb4a, Tardbp, Matr3, Rab1
12	Prion disease	2.07	Psmd2, Hspa1l, Psmc5, Psmc6, Csnk2b, Psmc2, Psmc4, Csnk2a2, Psmc3, Tubb4a
13	Purine metabolism	1.98	Adss, Adsl, Gart, Hprt, Paics, Impdh1, Impdh2
14	Spinocerebellar ataxia	1.91	Psmd2, Psmc5, Psmc6, Pum1, Psmc2, Psmc4, Psmc3
15	Alanine, aspartate and glutamate metabolism	1.88	Cad, Adss, Adsl, Asns
16	Adherens junction	1.84	Rhoa, Csnk2b, Csnk2a2, Mllt4, Iqgap1
17	Tight junction	1.75	Scrib, Rhoa, Pcna, Tjp2, Cttn, Amot, Mllt4
18	Biosynthesis of amino acids	1.69	Pfkl, Aldh18a1, Asns, Pfkm, Phgdh
19	Ribosome biogenesis in eukaryotes	1.69	Csnk2b, Fbl, Csnk2a2, Xpo1, Nop58
20	Metabolic pathways	1.45	Ckb, Gys1, Atp6v1b2, P4ha1, Cad, Adss, Pfkl, Adsl, Gart, Aldh18a1, Hprt, Ahcyl1, Ctps, Paics, Asns, Agl, Ptges3, Fasn, Pfkm, Ahcy, Phgdh, Pygl, Prdx6, Impdh1, Impdh2, Acly, Mthfd1l

Table S5. Top 20 Reactome pathways enriched in the C2C12 TAZ interactome dataset.

#	Reactome pathway – C2C12	-log (FDR)	Proteins
1	Metabolism of RNA	27.66	Chtop, Prpf6, U2af2, Psmd2, Ppp2r1a, Khsrp, Psmd3, Exosc10, Psmd11, Prpf8, Ywhab, Pelp1, Ltv1, Snrpf, Ppp2ca, Psmc5, Eftud2, Psmc1, Psmc6, Ywhaz, Prmt5, Etf1, Sf3b1, Psmc2, Polr2b, Srsf9, Psmc4, Xrn1, Fbl, Dhx9, Nop14, Rbm17, Srrt, Polr2l, Sf3b3, Dhx38, Exosc4, Patl1, Cpsf3, Psmc3, Snrnp70, Prpf40a, Sf3b4, Prpf38a, Cherp, U2surp, Gspt1, Rqcd1, Ppp2r2a, Snrnp200, Xpo1, Ppih, Adar, Snrpa1, Hspa1b, Ddx39b
2	Cell Cycle	15.25	Kpnb1, Tubb6, Rab8a, Psmd2, Ppp2r1a, Rpa3, Psmd3, Psmd11, Ywhab, Dync1h1, Ywhah, Cdk1, Ppp2ca, Rad50, Psmc5, Psmc1, Psmc6, Ywhaz, Csnk2b, Smc3, Psmc2, Ran, Ruvbl1, Psmc4, Dkc1, Mre11a, Smc1a, Tubg1, Nek9, Tuba1c, Ywhag, Mcm2, Ywhae, Psmc3, Tubb2b, Ppp2r2a, Hsp90aa1, Hdac1, Rbbp4, Xpo1, Ywhaq, Rab1

Journal of Cell Science • Supplementary information

3	Gene expression (Transcription)	14.74	Chtop, Paf1, U2af2, Ctr9, Psmd2, Ppp2r1a, Rpa3, Psmd3, Psmd11, Ywhab, Ywhah, Cdk1, Snrpf, Ppp2ca, Rad50, Psmc5, Psmc1, Psmc6, Ywhaz, Prmt5, Csnk2b, Sf3b1, Psmc2, Polr2b, Ran, Srsf9, Psmc4, Mre11a, Smarca4, Gtf3c3, Srrt, Polr2l, Dhx38, Ywhag, Gtf3c1, Tead1, Ep300, Cpsf3, Ywhae, Psmc3, Ssrp1, Polr1c, Rqcd1, Smarcc1, Usp9x, Mta2, Hdac1, Rbbp4, Ywhaq, Tead3, Arid1a, Usp7, Ddx39b, Tceb1
4	RNA Polymerase II Transcription	13.35	Chtop, Paf1, U2af2, Ctr9, Psmd2, Ppp2r1a, Rpa3, Psmd3, Psmd11, Ywhab, Ywhah, Cdk1, Snrpf, Ppp2ca, Rad50, Psmc5, Psmc1, Psmc6, Ywhaz, Prmt5, Csnk2b, Psmc2, Polr2b, Srsf9, Psmc4, Mre11a, Smarca4, Srrt, Polr2l, Dhx38, Ywhag, Tead1, Ep300, Cpsf3, Ywhae, Psmc3, Ssrp1, Rqcd1, Smarcc1, Usp9x, Mta2, Hdac1, Rbbp4, Ywhaq, Tead3, Arid1a, Usp7, Ddx39b, Tceb1
5	mRNA Splicing - Major Pathway	13.30	Prpf6, U2af2, Prpf8, Snrpf, Eftud2, Sf3b1, Polr2b, Srsf9, Dhx9, Rbm17, Srrt, Polr2l, Sf3b3, Dhx38, Cpsf3, Snrnp70, Prpf40a, Sf3b4, Prpf38a, Cherp, U2surp, Snrnp200, Ppih, Snrpa1
6	Processing of Capped Intron-Containing Pre-mRNA	12.87	Chtop, Prpf6, U2af2, Prpf8, Snrpf, Eftud2, Sf3b1, Polr2b, Srsf9, Dhx9, Rbm17, Srrt, Polr2l, Sf3b3, Dhx38, Cpsf3, Snrnp70, Prpf40a, Sf3b4, Prpf38a, Cherp, U2surp, Snrnp200, Ppih, Snrpa1, Ddx39b
7	G2/M Transition	12.41	Tubb6, Rab8a, Psmd2, Ppp2r1a, Psmd3, Psmd11, Dync1h1, Cdk1, Ppp2ca, Psmc5, Psmc1, Psmc6, Psmc2, Psmc4, Tubg1, Tuba1c, Ywhag, Ywhae, Psmc3, Tubb2b, Ppp2r2a, Hsp90aa1, Xpo1
8	Signaling by WNT	12.26	Psmd2, Ppp2r1a, Rhoa, Psmd3, Psmd11, Ap2b1, Ppp2ca, Psmc5, Psmc1, Psmc6, Ywhaz, Csnk2b, Psmc2, Psmc4, Vps35, Smarca4, Ep300, Psmc3, Rac1, Chd8, Hdac1, Xpo1, Clta, Tle3, Csnk1a1
9	Cell Cycle, Mitotic	11.91	Kpnb1, Tubb6, Rab8a, Psmd2, Ppp2r1a, Rpa3, Psmd3, Psmd11, Dync1h1, Cdk1, Ppp2ca, Psmc5, Psmc1, Psmc6, Csnk2b, Smc3, Psmc2, Ran, Psmc4, Smc1a, Tubg1, Nek9, Tuba1c, Ywhag, Mcm2, Ywhae, Psmc3, Tubb2b, Ppp2r2a, Hsp90aa1, Hdac1, Rbbp4, Xpo1, Rab1
10	G2/M Checkpoints	11.39	Psmd2, Rpa3, Psmd3, Psmd11, Ywhab, Ywhah, Cdk1, Rad50, Psmc5, Psmc1, Psmc6, Ywhaz, Psmc2, Psmc4, Mre11a, Ywhag, Mcm2, Ywhae, Psmc3, Ywhaq
11	M Phase	11.37	Kpnb1, Tubb6, Psmd2, Ppp2r1a, Psmd3, Psmd11, Dync1h1, Cdk1, Ppp2ca, Psmc5, Psmc1, Psmc6, Csnk2b, Smc3, Psmc2, Ran, Psmc4, Smc1a, Tubg1, Nek9, Tuba1c, Ywhag, Ywhae, Psmc3, Tubb2b, Ppp2r2a, Hsp90aa1, Xpo1, Rab1
12	Cellular responses to stress	11.35	Tubb6, Dnajb1, Psmd2, Hspa1l, Rpa3, Psmd3, Psmd11, Dync1h1, Rad50, Psmc5, Psmc1, Psmc6, Psmc2, Psmc4, Mre11a, Ccar2, Ptges3, Tuba1c, Ep300, Ywhae, Psmc3, Prdx6, Hsph1, Tubb2b, Hspa4l, Hsp90aa1, Rbbp4, St13, Hspa1b, Tceb1
13	TCF-dependent signaling in response to WNT	11.26	Psmd2, Ppp2r1a, Psmd3, Psmd11, Ppp2ca, Psmc5, Psmc1, Psmc6, Ywhaz, Csnk2b, Psmc2, Psmc4, Smarca4, Ep300, Psmc3, Chd8, Hdac1, Xpo1, Tle3, Csnk1a1
14	Regulation of mRNA stability by proteins that bind AU-rich elements	10.77	Psmd2, Khsrp, Psmd3, Psmd11, Ywhab, Psmc5, Psmc1, Psmc6, Ywhaz, Psmc2, Psmc4, Xrn1, Exosc4, Psmc3, Xpo1, Hspa1b
15	Immune System	10.62	Actr2, Ilf2, Kpnb1, Npepps, Tubb6, Ap1m1, Ube2m, Arpc2, Psmd2, Ppp2r1a, Rhoa, Psmd3, Psmd11, Ywhab, Dync1h1, Ap2b1, Rab5c, Cand1, Pfkl, Ppp2ca, Psmc5, Sec23a, Psmc1, Ddx41, Psmc6, Ywhaz, Csnk2b, Huwe1, Sec22b, Psmc2, Cyfip1, Psmc4, Copb1, Mre11a, Smarca4, Pkm, Dhx9, Sec24c, Eef2, Tuba1c, Rap1b, Ep300, Psmc3, Pygl, Prdx6, Tubb2b, Rac1, Impdh2, Arf1, Ube2o, Aldoa, Hsp90aa1, Sec31a, Ubr4, Acly, Clta, Eif4g2, Dnm2, Hspa1b, Tceb1
16	Cell Cycle Checkpoints	10.38	Psmd2, Ppp2r1a, Rpa3, Psmd3, Psmd11, Ywhab, Dync1h1, Ywhah, Cdk1, Ppp2ca, Rad50, Psmc5, Psmc1, Psmc6, Ywhaz, Psmc2, Psmc4, Mre11a, Ywhag, Mcm2, Ywhae, Psmc3, Xpo1, Ywhaq
17	Mitotic Anaphase	10.17	Kpnb1, Tubb6, Psmd2, Ppp2r1a, Psmd3, Psmd11, Dync1h1, Cdk1, Ppp2ca, Psmc5, Psmc1, Psmc6, Smc3, Psmc2, Ran, Psmc4, Smc1a, Tuba1c, Psmc3, Tubb2b, Ppp2r2a, Xpo1

Journal of Cell Science • Supplementary information

18	Innate Immune System	9.77	Actr2, Ilf2, Kpnb1, Ap1m1, Ube2m, Arpc2, Psmd2, Ppp2r1a, Rhoa, Psmd3, Psmd11, Dync1h1, Rab5c, Cand1, Pfkl, Ppp2ca, Psmc5, Psmc1, Ddx41, Psmc6, Csnk2b, Huwe1, Psmc2, Cyfip1, Psmc4, Copb1, Mre11a, Pkm, Dhx9, Eef2, Rap1b, Ep300, Psmc3, Pygl, Prdx6, Rac1, Impdh2, Aldoa, Hsp90aa1, Ubr4, Acly, Dnm2, Hspa1b
19	Generic Transcription Pathway	9.71	Psmd2, Ppp2r1a, Rpa3, Psmd3, Psmd11, Ywhab, Ywhah, Cdk1, Ppp2ca, Rad50, Psmc5, Psmc1, Psmc6, Ywhaz, Prmt5, Csnk2b, Psmc2, Polr2b, Psmc4, Mre11a, Smarca4, Polr2l, Ywhag, Tead1, Ep300, Ywhae, Psmc3, Ssrp1, Rqcd1, Smarcc1, Usp9x, Mta2, Hdac1, Rbbp4, Ywhaq, Tead3, Arid1a, Usp7, Tceb1
20	Transcriptional Regulation by TP53	9.53	Ppp2r1a, Rpa3, Ywhab, Ywhah, Cdk1, Ppp2ca, Rad50, Ywhaz, Prmt5, Csnk2b, Polr2b, Mre11a, Polr2l, Ywhag, Ep300, Ywhae, Ssrp1, Rqcd1, Mta2, Hdac1, Rbbp4, Ywhaq, Usp7, Tceb1

Table S6. Top 20 Reactome pathways enriched in the NRCM TAZ interactome dataset.

#	Reactome pathways – NRVM	-log(FDR)	Proteins
1	Metabolism of RNA	25.39	Prpf6, U2af2, Bcas2, Psmd2, Rplp1, U2af1, Prpf8, Ywhab, Ftsj3, Psmc5, Eftud2, Skiv2l2, Psmc6, Ywhaz, Prmt5, Sf3b2, Alyref, Eif4a3, Sf3b1, Psmc2, Dhx15, Polr2b, Ddx47, Psmc4, Fbl, Dhx9, Edc4, Rbm17, Dis3, Dhx38, Poldip3, Psmc3, Cpsf1, U2surp, Gspt1, Srrm2, Cnot1, Snrnp200, Xpo1, Fip1l1, Rpl39, Plrg1, Rnps1, Ddx39b, Prpf19, Hnrnpr, Nop58
2	mRNA Splicing - Major Pathway	17.01	Prpf6, U2af2, Bcas2, U2af1, Prpf8, Eftud2, Skiv2l2, Sf3b2, Alyref, Eif4a3, Sf3b1, Dhx15, Polr2b, Dhx9, Rbm17, Dhx38, Cpsf1, U2surp, Srrm2, Snrnp200, Fip1l1, Plrg1, Rnps1, Prpf19, Hnrnpr
3	Processing of Capped Intron-Containing Pre-mRNA	16.82	Prpf6, U2af2, Bcas2, U2af1, Prpf8, Eftud2, Skiv2l2, Sf3b2, Alyref, Eif4a3, Sf3b1, Dhx15, Polr2b, Dhx9, Rbm17, Dhx38, Poldip3, Cpsf1, U2surp, Srrm2, Snrnp200, Fip1l1, Plrg1, Rnps1, Ddx39b, Prpf19, Hnrnpr
4	RNA Polymerase II Transcription	12.98	U2af2, Trim28, Psmd2, U2af1, Ywhab, Ywhah, Psmc5, Psmc6, Ywhaz, Smarcd1, Prmt5, Csnk2b, Alyref, Eif4a3, Parp1, Pcna, Psmc2, Polr2b, Cbx3, Psmc4, Rbbp7, Smarca4, Supt16, Zfhx3, Dhx38, Ywhag, Poldip3, Csnk2a2, Chd4, Ywhae, Psmc3, Cpsf1, Ssrp1, Smarcc1, Mta2, Cnot1, Prmt1, Fip1l1, Arid1a, Usp7, Rnps1, Ddx39b
5	Gene expression (Transcription)	12.96	U2af2, Trim28, Psmd2, U2af1, Ywhab, Ywhah, Psmc5, Psmc6, Ywhaz, Smarcd1, Prmt5, Csnk2b, Alyref, Eif4a3, Sf3b1, Parp1, Pcna, Psmc2, Polr2b, Cbx3, Psmc4, Rbbp7, Smarca4, Supt16, Smarca5, Zfhx3, Dhx38, Ywhag, Poldip3, Csnk2a2, Chd4, Ywhae, Psmc3, Cpsf1, Ssrp1, Smarcc1, Mta2, Cnot1, Prmt1, Fip1l1, Arid1a, Usp7, Rnps1, Ddx39b
6	Cell Cycle	12.86	Mcm7, Kpnb1, Psmd2, Ywhab, Ywhah, Psmc5, Psmc6, Ywhaz, Mcm4, Csnk2b, Smc3, Mcm6, Pcna, Psmc2, Psmc4, Rbbp7, Prkar2b, Smarca5, Smc1a, Smc4, Ywhag, Csnk2a2, Mcm3, Mcm2, Ywhae, Psmc3, Tubb4a, Rcc2, Hsp90aa1, Xpo1, Smc2, Mcm5, Rab1
7	Cell Cycle, Mitotic	10.57	Mcm7, Kpnb1, Psmd2, Psmc5, Psmc6, Mcm4, Csnk2b, Smc3, Mcm6, Pcna, Psmc2, Psmc4, Prkar2b, Smc1a, Smc4, Ywhag, Csnk2a2, Mcm3, Mcm2, Ywhae, Psmc3, Tubb4a, Rcc2, Hsp90aa1, Xpo1, Smc2, Mcm5, Rab1
8	G2/M Checkpoints	10.34	Mcm7, Psmd2, Ywhab, Ywhah, Psmc5, Psmc6, Ywhaz, Mcm4, Mcm6, Psmc2, Psmc4, Ywhag, Mcm3, Mcm2, Ywhae, Psmc3, Mcm5
9	Metabolism of proteins	9.32	Ube2m, Psmd2, Rhoa, Rplp1, Srp14, Rab5c, Cand1, Psmc5, Scfd1, Psmc6, Eif3e, Prkdc, Mrpl40, Csnk2b, Otub1, Smc3, Eif3a, Parp1, Fbn1, Pcna, Psmc2, Cct6a, Gnb2, Psmc4, Eif3c, Copb1, Eif3f, Rbbp7, Arcn1, Mrps22, Sec23ip, Smc1a, Mrps7, Csnk2a2, Psmc3, Tubb4a, Gfm1, Gspt1, Eif3d, Rab7, Copg1, Rpl39, Usp10, Usp7, Pcmt1, Rab1, Mrpl16, Nop58
10	Assembly of the pre-replicative complex	8.73	Mcm7, Psmd2, Psmc5, Psmc6, Mcm4, Mcm6, Psmc2, Psmc4, Mcm3, Mcm2, Psmc3, Mcm5
11	Orc1 removal from chromatin	8.57	Mcm7, Psmd2, Psmc5, Psmc6, Mcm4, Mcm6, Psmc2, Psmc4, Mcm3, Mcm2, Psmc3, Mcm5

Journal of Cell Science • Supplementary information

12	Cell Cycle Checkpoints	8.32	Mcm7, Psmd2, Ywhab, Ywhah, Psmc5, Psmc6, Ywhaz, Mcm4, Mcm6, Psmc2, Psmc4, Ywhag, Mcm3, Mcm2, Ywhae, Psmc3, Rcc2, Xpo1, Mcm5
13	Metabolism of RNA	8.24	Mcm7, Psmd2, Psmc5, Psmc6, Mcm4, Smc3, Mcm6, Pcna, Psmc2, Psmc4, Smc1a, Mcm3, Mcm2, Psmc3, Mcm5
14	mRNA Splicing - Major Pathway	8.22	Trim28, Psmd2, Ywhab, Ywhah, Psmc5, Psmc6, Ywhaz, Smarcd1, Prmt5, Csnk2b, Parp1, Pcna, Psmc2, Polr2b, Cbx3, Psmc4, Rbbp7, Smarca4, Supt16, Zfhx3, Ywhag, Csnk2a2, Chd4, Ywhae, Psmc3, Ssrp1, Smarcc1, Mta2, Cnot1, Prmt1, Arid1a, Usp7
15	Processing of Capped Intron-Containing Pre-mRNA	7.83	Kpnb1, Psmd2, Psmc5, Psmc6, Csnk2b, Smc3, Psmc2, Psmc4, Prkar2b, Smc1a, Smc4, Ywhag, Csnk2a2, Ywhae, Psmc3, Tubb4a, Rcc2, Hsp90aa1, Xpo1, Smc2, Rab1
16	RNA Polymerase II Transcription	7.45	Mcm7, Psmd2, Psmc5, Psmc6, Mcm4, Mcm6, Pcna, Psmc2, Psmc4, Mcm3, Mcm2, Psmc3, Mcm5
17	Gene expression (Transcription)	7.39	Kpnb1, Ap1m1, Psmd2, Rhoa, Srp14, Rab5c, Cand1, Pfkl, Csnk2b, Capn1, Psmc2, Cyfip1, Copb1, Agl, Psmc3, Pygl, Prdx6, Impdh1, Impdh2, Hsp90aa1, Ubr4, Acly, Rab7, Iqgap1
18	Cell Cycle	7.39	U2af2, U2af1, Alyref, Eif4a3, Dhx38, Poldip3, Cpsf1, Fip1l1, Rnps1, Ddx39b
19	Cell Cycle, Mitotic	7.15	Cad, Adss, Adsl, Gart, Paics, Impdh1, Impdh2
20	G2/M Checkpoints	6.99	Mcm7, Psmd2, Psmc5, Psmc6, Mcm4, Mcm6, Psmc2, Psmc4, Mcm3, Mcm2, Psmc3, Mcm5

Journal of Cell Science • Supplementary information
