# Supplementary material for: TAZ interactome analysis using nanotrap-based affinity purification–mass spectrometry
Source: J Cell Sci. 2025 Feb 24;138(4):jcs263527. doi: 10.1242/jcs.263527 (PMC11928053; doi:10.1242/jcs.263527)
Supplement: Supplementary file 1 [file joces-138-263527_review_history.pdf]

Journal of Cell Science

TAZ interactome analysis using nanotrap-based affinity purification-mass spectrometry

Jonathan Kelebeev, Anastasia MacKeracher, Tetsuaki Miyake and John C. McDermott
DOI: 10.1242/jcs.263527

Editor: Robert Parton

Review timeline

Original submission:	5 September 2024
Editorial decision:	3 October 2024
First revision received:	10 December 2024
Editorial decision:	24 December 2024
Second revision received:	13 January 2025
Accepted:	15 January 2025

Original submission

First decision letter

MS ID#: jcs.263527

MS TITLE: TAZ (WWTR1) Interactome Analysis using a GBP nanotrap based affinity purification approach

AUTHORS: John C. McDermott; Jonathan Kelebeev; Anastasia MacKeracher; Tetsuaki Miyake

ARTICLE TYPE: Tools and Resources

Dear Dr McDermott,

We have now reached a decision on the above manuscript.

As you will see, the reviewers raise a number of substantial criticisms that prevent me from accepting the paper at this stage. They suggest, however, that a revised version might prove acceptable, if you can address their concerns. If you think that you can deal satisfactorily with the criticisms on revision, I would be pleased to see a revised manuscript. We would then return it to the reviewers.

Reviewer 1

Advance summary and potential significance to field

Kelebeev et al. report on a TAZ protein interactome analysis in C2C12 skeletal myoblasts and primary rat cardiomyocytes. Many proteins were newly identified as TAZ interaction partners. To validate the analysis, the authors focused on one such binding protein, CARM1 (a methyltransferase). TAZ and CARM1 co-IP in transfectants, and co-expression of CARM1 with TAZ neutralizes TAZ-mediated HOP reporter activity in 293 cells. CARM1 methylates TAZ on R77, which

© 2025. Published by The Company of Biologists under the terms of the Creative Commons Attribution License (https://creativecommons.org/licenses/by/4.0/).

appears to promote phosphorylation at the known site, S89, which in turn leads to cytoplasmic localization.

This manuscript reports a useful interactome approach and analysis, leading to a previously unobserved interaction between TAZ and CARM1. The interactome analysis is a strength of the paper, and the TAZ-CARM1 interaction is an interesting finding. The analysis of that interaction and its consequences also, however, contains weaknesses that should be addressed with additional information and experimentation.

Comments for the author

1. Figure 3A: The authors conclude that there is an increase in the cytoplasmic localization of TAZ in C2C12 cells after 96 hours in DM. In discussing this figure on page 8, they inadvertently failed to remove a parenthetical comment that must have been a way of communicating between themselves during manuscript preparation ("weak argument due to no clear evidences"). I agree with the comment - the results shown in Figure 3A are not a convincing demonstration of increased TAZ cytoplasmic localization under that condition. If the authors suspect that this is true, they need to provide convincing, quantitative data to support their contention. Otherwise, the sentence (and the comment) should be removed.

2. For many figure panels, there is little or no mention of how many times the experiment was repeated or actual quantification of data when quantitative conclusions are being drawn. It is essential that this information be included throughout. Among these are:

A. Figure 3D: What do the individual data points represent? What is the true N for these experiments?

B. For Figures 3E, 4A, and 4B: The Western blots are all discussed in a quantitative manner but no densitometric analysis or normalization to controls was performed, nor was the number of biological replicates reported. Also, what do the asterisks in Figures 4A and 4B indicate? I initially thought this might be heavy chain but 4A is a straight WB. Again, no N value is reported.

3. Figure 4B (right panel): This figure purports to show that CARM1 methylates TAZ. Co-expression of CARM1 with TAZ leads to a band recognized by mono-/di-methyl antibody. Is it possible that some or all of this band could be auto-methylated CARM1? CARM1 is known to have automethylation activity. The band is in the size range shown here for both TAZ and CARM1, and no CARM1 IP/methyl WB control was performed. Additionally, Figures 4D and 4E purport to provide a direct demonstration of CARM1-dependent TAZ methylation and identification of the site. The actual MS data should be provided as a supplemental figure.

4. Figure S2 reports on the rhabdomyosarcoma cell line, RD. It is not at all clear what the authors are seeking to show here, and the experiments are very preliminary. This supplemental figure does not add anything useful to the paper, and I recommend it be removed.

Minor points:

1. In Figure 1, the font size in most panels is too small to be readable.

2. Pg. 7: It is stated that "…CARM1 and TAZ were co-localized in the nucleus of C2C12 myoblasts (Figure 3E)". The resolution of the figure really only supports the conclusion that "CARM1 and TAZ were each localized in the nucleus". Actual co-localization within the nucleus is not clear.

<u>Reviewer 2</u>

Advance summary and potential significance to field

© 2025. Published by The Company of Biologists under the terms of the Creative Commons Attribution License (https://creativecommons.org/licenses/by/4.0/).

The authors use a nanotrap-based affinity purification approach with mass spectrometry protein identification to identify TAZ-interacting proteins in myogenic cells. This approach has the potential to make new discoveries in an important signalling pathway. While the techniques on their own are not novel, the application used in this manuscript allows the authors to circumvent the need for ectopic overexpression in difficult to transfect cells.

Comments for the author

Major comments/concerns:

1. With regards to the production of bait protein isolated by transfection of HEK293T cells, can authors provide evidence that there is no carryover protein from HEK293T cells (other than EYFP-TAZ)? An ideal negative control would have been bait protein in the absence of prey protein lysate.

2. How many replicates of affinity purification and mass spectrometry were performed?

3. How does this method compare to other established methods for interactome discovery, eg using GST-tagged purified protein as bait? As this is a Tools/Resource article, advantages (as well as potential caveats) in comparison to existing systems should be discussed.

4. Methods - specific details of NP40 lysis buffer (page 17) used to isolate bait protein should be given to allow the method to be reproduced by others.

5. For all western blots: uncropped blots should be shown in the supplementary material.

6. Page 8, line 25/26: "TAZ exhibited an increase in cytoplasmic localization in the DM 96 hour condition compared to the proliferative (GM) state". This conclusion is difficult to assess without extra experiments such as fractionation.

7. Fig 3A, S2A, 4A - labels in figures indicate that the antibody recognises YAP/TAZ, although only TAZ is mentioned in the results text. There are also two bands in fig 4A - do these correspond to YAP and TAZ? If so, could authors clarify if the immunofluorescence in 3A and S2A is specific for TAZ?

8. Fig 3E - Decrease in myogenin protein levels in cells treated with CARM1 inhibitor. Actin loading also looks decreased - this should be quantitated.

Minor comments:

1. Page 5, line 24/25: "Based on our previous identification of TAZ as a novel repressor of skeletal muscle". There should be a reference here.

2. Page 15 - use of 2% FBS for differentiation of C2C12 cells. 2% horse serum is the standard for differentiation of C2C12 cells. Can authors comment on why they used FBS instead?

3. Details of CARM1 inhibitor should be included in methods section.

4. Quantitation in 2F, 3B, 3D: It should be specified whether the datapoints representative of technical replicates or independent experiments.

5. Fig 2F, 3B, S2B - "-" symbols are missing in graph.

5. "GBP" abbreviation in title should be avoided.

First revision

© 2025. Published by The Company of Biologists under the terms of the Creative Commons Attribution License (https://creativecommons.org/licenses/by/4.0/).

Author response to reviewers' comments

Reviewer 1:

1. With regards to the production of bait protein isolated by transfection of HEK293T cells, can authors provide evidence that there is no carryover protein from HEK293T cells (other than EYFP-TAZ)? An ideal negative control would have been bait protein in the absence of prey protein lysate.

RESPONSE: To control for possible carryover of protein from HEK293T cells into the affinity trap, the bait- bead complex undergoes extensive washing with a harsh wash buffer, such as RIPA buffer. Extensive washes of the bait are possible because of the high affinity of EYFP with the GBP nanobodies localized on the beads. Additionally, species-specific protein reference databases are used in the mass spectrometry pipeline—mouse for C2C12 samples, rat for cardiomyocyte samples and human for HEK293T cells—to identify the proteins. Thus, even if there are trace proteins from HEK 293T cells detected in the mass spectrometry, they are removed in the analysis pipeline due to being of Human origin.

2. How many replicates of affinity purification and mass spectrometry were performed?

RESPONSE: Two replicates each for C2C12, cardiomyocytes and control EYFP were performed. This is now documented in the figure 1 caption.

3. How does this method compare to other established methods for interactome discovery, eg using GST- tagged purified protein as bait? As this is a Tools/Resource article, advantages (as well as potential caveats) in comparison to existing systems should be discussed.

RESPONSE: We agree that indicating some of the advantages of this method over other interactome methods is reasonable although an in depth discussion of the many other types of protein interaction technologies would be quite unwieldy since there are so many of them. We have however included the following text in the discussion focusing on some of the advantages and possible disadvantages of our method compared to some approaches in the literature:

This affinity capture method used here offers a number of advantages for interactome analysis. First, this approach can be applied to model systems that are difficult to transfect, such as primary cells (cardiomyocytes being particularly difficult). By using an easy-to-transfect cell line like HEK293T, robust expression and purification of the bait protein is achieved, bypassing transfection challenges associated with expressing tagged proteins in the cells of interest. Unlike other proteomic interactome workflows, this method therefore avoids ectopic expression of the bait protein in the cells of interest, which can lead to stoichiometry issues, alteration of the target cell proteome and non-physiological interactions due to ectopic overexpression in the target cells. The approach reported herein thus maintains the unaltered and native state of the endogenous proteome in the prey protein lysate (target cells). Lastly, the flexibility of this approach enables interactome screenings across diverse biological systems using a consistent bait protein. This standardization simplifies comparisons of interactomes from different cell types, as the bait protein remains constant, minimizing variability. This is particularly beneficial for comparative studies, high-throughput screens, and exploring how interactions vary across different cell types or experimental conditions. In terms of caveats, it should be noted that the bait protein is produced in HEK293T cells and may not be post-translationally modified in the same manner as the native protein in the target cell prey lysate. This could lead to the identification of non-physiological interactions. However, production of the bait protein in HEK293T cells minimally maintains normal mammalian cell processing and basal modification of the protein, which one might consider an advantage over using bacterially expressed proteins (eg GST tagged) as bait which would not be processed or modified in a similar manner. Obviously there are pros and cons of many interactome methods, therefore necessitating careful further validation of interactions using co-precipitation and functional assays.

© 2025. Published by The Company of Biologists under the terms of the Creative Commons Attribution License (https://creativecommons.org/licenses/by/4.0/).

4. Methods - specific details of NP40 lysis buffer (page 17) used to isolate bait protein should be given to allow the method to be reproduced by others.

RESPONSE: Thank you- this has been added to the methods as suggested- 150mM NaCl, 1.0% (V/V) NP-40, 50mM Tris (pH8.0), and inhibitors.

5. For all western blots: uncropped blots should be shown in the supplementary material.

RESPONSE: This has now been **added** – supplemental Figure 3

6. Page 8, line 25/26: "TAZ exhibited an increase in cytoplasmic localization in the DM 96 hour condition compared to the proliferative (GM) state". This conclusion is difficult to assess without extra experiments such as fractionation.

RESPONSE: We agree with the reviewer so we did some additional experimentation and quantitation to further address this issue and are now confident, based on this analysis, that there is an increase in TAZ cytoplasmic sequestration in DM conditions. See figure 3A and Figure S2.

7. Fig 3A, S2A, 4A - labels in figures indicate that the antibody recognises YAP/TAZ, although only TAZ is mentioned in the results text. There are also two bands in fig 4A - do these correspond to YAP and TAZ? If so, could authors clarify if the immunofluorescence in 3A and S2A is specific for TAZ?

RESPONSE: The antibody used in this study recognizes a slow-migrating (YAP) and a faster-migrating (TAZ) band. We have clarified which band corresponds to TAZ by the addition of an asterisk. The caption also reflects this change.

8. Fig 3E - Decrease in myogenin protein levels in cells treated with CARM1 inhibitor. Actin loading also looks decreased - this should be quantitated.

RESPONSE: **We did go back and quantitate the Myogenin level with and without the CARM1 inhibitor nornmalized for the Actin level. The result is shown below. We did not include this additional documentation in the manuscript since the effects of Carm1 on myogenesis is a peripheral aspect of our manuscript. Other studies have shown a general effect of Carm1 on myogenesis. Of course this is a sum of the effect on all Carm1 targets and doesn't necessarily address the TAZ/Carm1 interaction specifically.**

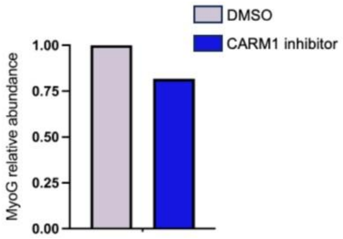

Minor comments:

1. Page 5, line 24/25: "Based on our previous identification of TAZ as a novel repressor of skeletal muscle". There should be a reference here.

RESPONSE: Thanks for pointing this out. We have now added this reference to our previous work.

2. Page 15 - use of 2% FBS for differentiation of C2C12 cells. 2% horse serum is the standard for differentiation of C2C12 cells. Can authors comment on why they used FBS instead?

RESPONSE: We have been working with C2C12 for over 30 years and have found that the cells

© 2025. Published by The Company of Biologists under the terms of the Creative Commons Attribution License (https://creativecommons.org/licenses/by/4.0/).

differentiate at approximately the same rate in 2% FBS compared to Horse serum and are somewhat healthier, producing larger and more robust multinucleated myotubes upon differentiation.

3. Details of CARM1 inhibitor should be included in methods section.

RESPONSE: Thanks for pointing this out – this has now been added to the Methods.

4. Quantitation in 2F, 3B, 3D: It should be specified whether the datapoints representative of technical replicates or independent experiments.

RESPONSE: We have now clarified this in the related Figure captions as follows:
Fig 2F – datapoints represent independent biological replicates
(luciferase)
Fig 3B (now 3C) – data points represent independent biological replicates (luciferase)
Fig 3D (now 3E) – data points represent technical replicates

5. Fig 2F, 3B, S2B - "-" symbols are missing in graph.

RESPONSE: "-" ***thanks -symbols have now been added.***

5. "GBP" abbreviation in title should be avoided.

RESPONSE: Agreed- we realized that we can take GBP out of the title completely since this states the specific nanobody used and that's not really necessary in the title. Spelling out GBP in its entirety would also make the title too wordy.
The title now reads:

TAZ (WWTR1) Interactome Analysis using nanotrap based a<inity purification- mass spectrometry

Reviewer 2:

1. Figure 3A: The authors conclude that there is an increase in the cytoplasmic localization of TAZ in C2C12 cells after 96 hours in DM. In discussing this figure on page 8, they inadvertently failed to remove a parenthetical comment that must have been a way of communicating between themselves during manuscript preparation ("weak argument due to no clear evidences"). I agree with the comment - the results shown in Figure 3A are not a convincing demonstration of increased TAZ cytoplasmic localization under that condition. If the authors suspect that this is true, they need to provide convincing, quantitative data to support their contention. Otherwise, the sentence (and the comment) should be removed.

RESPONSE: As documented above in response to Reviewer 1, we did some additional experimentation and quantitation to further address this issue and are now confident, based on this analysis, that there is an increase in TAZ cytoplasmic sequestration in DM conditions. See figure 3A and Figure S2.

2. For many figure panels, there is little or no mention of how many times the experiment was repeated or actual quantification of data when quantitative conclusions are being drawn. It is essential that this information be included throughout. Among these are:

A. Figure 3D: What do the individual data points represent? What is the true N for these experiments?

RESPONSE: The datapoints in Figure 3E (formerly Figure 3D) represent the average number of cells in the field of view of the image.

B. For Figures 3E, 4A, and 4B: The Western blots are all discussed in a quantitative manner but no densitometric analysis or normalization to controls was performed, nor was the number of biological replicates reported. Also, what do the asterisks in Figures 4A and 4B

© 2025. Published by The Company of Biologists under the terms of the Creative Commons Attribution License (https://creativecommons.org/licenses/by/4.0/).

indicate? I initially thought this might be heavy chain but 4A is a straight WB. Again, no N value is reported.

RESPONSE:
We have now clarified issues related to this point in the related Figure captions as follows:
Fig 2F – datapoints represent independent biological replicates (luciferase)
Fig 3B – data points represent independent biological replicates(luciferase)
Fig 3D (now 3E) – data points represent technical replicates

The antibody used in this study recognizes a slower-migrating (YAP) and a faster-migrating (TAZ) band. We have clarified which band corresponds to TAZ by the addition of an asterisk. The caption also reflects this change.

3. Figure 4B (right panel): This figure purports to show that CARM1 methylates TAZ. Co-expression of CARM1 with TAZ leads to a band recognized by mono-/di-methyl antibody. Is it possible that some or all of this band could be auto-methylated CARM1? CARM1 is known to have automethylation activity. The band is in the size range shown here for both TAZ and CARM1, and no CARM1 IP/methyl WB control was performed. Additionally, Figures 4D and 4E purport to provide a direct demonstration of CARM1- dependent TAZ methylation and identification of the site. The actual MS data should be provided as a supplemental figure.

RESPONSE: The figure depicts a FLAG IP of an eluate that came from a lysate that had FLAG-TAZ overexpressed. We also show in the supplemental figs and inserted below that FLAG-TAZ (or HA-TAZ) and HA-CARM1 have differing mobilities in SDS PAGE.

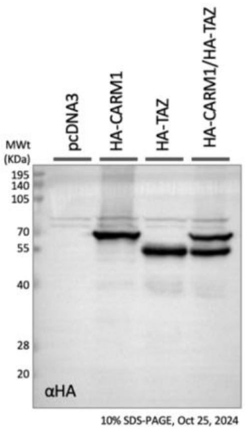

The m-s identification of TAZ methylation is the foundation of a further more expansive mechanistic study documenting the impact of di-methylation on a variety of TAZ functions. Since the current manuscript is mainly focused on the utilization of the GBP nanotrap methodology as a "Tools and Resource" paper, we do not want to include the highly detailed examination of the TAZ methylation landscape since it would, to some degree, divert the main them of the manuscript. However, we would point out that the intensity scores for the unmthylated and methylated (R77) forms of TAZ in Fig 4E are direct M-S data from the TIMS Tof analysis. For the purpose of the reviewers, we would like to indicate the type of M/Z data utilized in this analysis as follows which denotes the peptide peaks corresponding to the peptide of interest on TAZ +/- Carm1 (the peaks on the lower Spectra indicate the unmodified and modified (mono and di-methylated) peptides: the unmodified and mono-methylated peaks are present in the absence of Carm1, and the additional di-methylated peptide is only present with Carm 1 co- expression (Carm 1 is an argine dimethyltransferase. Note that the peptides here are triply charged and the intensity scores on the Y axis depicts the M-S intensity score x 10^4.

© 2025. Published by The Company of Biologists under the terms of the Creative Commons Attribution License (https://creativecommons.org/licenses/by/4.0/).

TAZ alone spectra

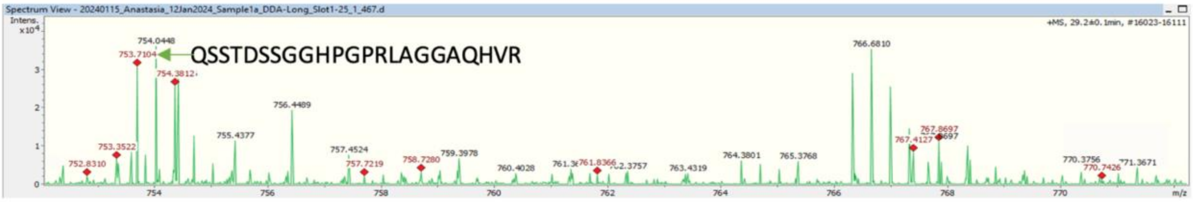

TAX + CARM1 spectra

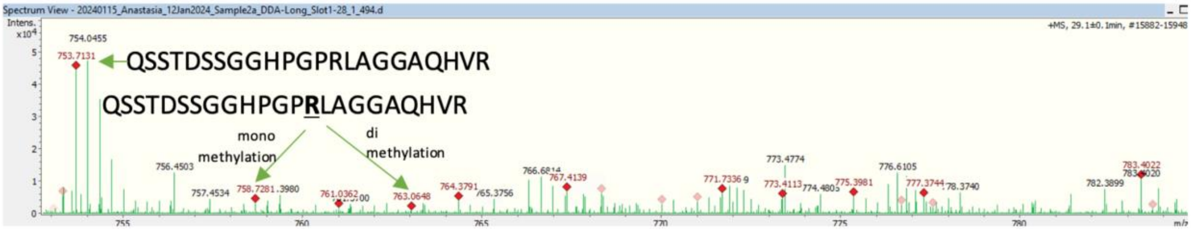

4. Figure S2 reports on the rhabdomyosarcoma cell line, RD. It is not at all clear what the authors are seeking to show here, and the experiments are very preliminary. This supplemental figure does not add anything useful to the paper, and I recommend it be removed.

RESPONSE: We agree with the reviewers point and have now removed all RMS data and corresponding mention of it in the text

Minor points:

1. In Figure 1, the font size in most panels is too small to be readable.

RESPONSE: We have now enhanced the size and quality of these images.

2. Pg. 7: It is stated that "...CARM1 and TAZ were co-localized in the nucleus of C2C12 myoblasts (Figure 3E)". The resolution of the figure really only supports the conclusion that "CARM1 and TAZ were each localized in the nucleus". Actual co-localization within the nucleus is not clear.

RESPONSE:We have now paid careful attention to the semantic issues here as pointed out by the reviewer. The text now states : "CARM1 and TAZ were each localized in the nucleus."

Second decision letter

MS ID#: jcs.263527R1

MS TITLE: TAZ (WWTR1) Interactome Analysis using nanotrap based affinity purification-mass spectrometry

AUTHORS: John C. McDermott; Jonathan Kelebeev; Anastasia MacKeracher; Tetsuaki Miyake

ARTICLE TYPE: Tools and Resources

Dear Dr McDermott,

We have now reached a decision on the above manuscript.

© 2025. Published by The Company of Biologists under the terms of the Creative Commons Attribution License (https://creativecommons.org/licenses/by/4.0/).

As you will see, the reviewers gave favourable reports but still raised some critical points that will require amendments to your manuscript as they were not satisfactorily addressed in your revision. I hope that you will be able to fully address all these comments in your revised manuscript so that we can proceed to publication.

Reviewer 1

Advance summary and potential significance to field

Comments for the author

Most of my comments have been addressed in a satisfactory manner. However, in response to my comment 2 about what data points mean and what the N is for each experiment, the response is not clear and at odds with the changes to the manuscript. The response states:

Fig 2F - datapoints represent independent biological replicates (luciferase)
Fig 3B - data points represent independent biological replicates(luciferase)
Fig 3D (now 3E) - data points represent technical replicates

In fact the figure legends for 2F, 3C (not 3B, this must be an error), and 3D all say the points represent technical replicates from an "individual (or "one") biological sample". What does this actually mean? Transfections were performed on three plates of the same cells in the same experiment? One plate with three assays? It is generally accepted that the number of biological replicates constitutes the N for the experiment. Unless these experiments have been performed in a way I don't understand, they therefore have N=1. N should equal at least 3.
> This appears to address the wrong figures highlighted by Reviewer #2, and clarification regarding quantitation and number of replicates is required.

Second revision

Author response to reviewers' comments

The authors have addressed most of the reviewers comments/suggestions, including additional text for clarification, and extra experiments/quantitation (figure 3A and Figure S2).However, there were some instances where the reviewers comments have not yet been directly addressed and these are outlined below.

Reviewer #1, point 7:
7. Fig 3A, S2A, 4A - labels in figures indicate that the antibody recognises YAP/TAZ, although only TAZ is mentioned in the results text. There are also two bands in fig 4A - do these correspond to YAP and TAZ? If so, could authors clarify if the immunofluorescence in 3A and S2A is specific for TAZ?
PREVIOUS RESPONSE: The antibody used in this study recognizes a slow-migrating (YAP) and a faster-migrating (TAZ) band. We have clarified which band corresponds to TAZ by the addition of an asterisk. The caption also reflects this change.

> Please address the question regarding the immunofluorescence specificity. If it recognises both YAP/TAZ, this should be clarified in the main text as well.

Author's response: On Figs 2E, 3A, S2A (Supp2B in most recent revision) the IF figures are actually labelled as YAP/TAZ due to the fact that, as correctly indicated by the reviewer, the antibody recognizes both. However, we had shown in a previous manuscript (Tripathi et al) that YAP is expressed at low levels compared to TAZ in myogenic cells (which is why we have focussed our

© 2025. Published by The Company of Biologists under the terms of the Creative Commons Attribution License (https://creativecommons.org/licenses/by/4.0/).

myogenic studies on TAZ). However, we see the reviewer's point that we should state both YAP/TAZ in the manuscript text when referring to studies using this antibody on immunofluorescence (in Westerns it is possible to distinguish them due to their Mr diYerences). We have now referred to this in the text as suggested.

Reviewer #1, point 8:
8. Fig 3E - Decrease in myogenin protein levels in cells treated with CARM1 inhibitor. Actin loading also looks decreased - this should be quantitated.
RESPONSE: We did go back and quantitate the Myogenin level with and without the CARM1 inhibitor normalized for the Actin level. The result is shown below. We did not include this additional documentation in the manuscript since the eJects of Carm1 on myogenesis is a peripheral aspect of our manuscript. Other studies have shown a general eJect of Carm1 on myogenesis. Of course this is a sum of the eJect on all Carm1 targets and doesn't necessarily address the TAZ/Carm1 interaction specifically.
> While the finding may be a peripheral aspect, reviewer #2 made a similar comment regarding quantitation. Authors should at least tone down their conclusion in the manuscript ("Western blot analysis confirmed a decrease in Myogenin protein levels in C2C12 cells treated with the CARM1 inhibitor compared to the DMSO control") given the quantitation is based on a single biological replicate and is not presented.

Author's response: We agree with toning down this point and, as suggested, have now included the following statement: Western blot analysis indicated a decrease in Myogenin protein levels in C2C12 cells treated with the CARM1 inhibitor compared to the DMSO control.

Reviewer #2, point 2B:
B. For Figures 3E, 4A, and 4B: The Western blots are all discussed in a quantitative manner but no densitometric analysis or normalization to controls was performed, nor was the number of biological replicates reported. Also, what do the asterisks in Figures 4A and 4B indicate? I initially thought this might be heavy chain but 4A is a straight WB. Again, no N value is reported.
RESPONSE:
We have now clarified issues related to this point in the related Figure captions as follows: Fig 2F - datapoints represent independent biological replicates (luciferase)
Fig 3B - data points represent independent biological replicates(luciferase)
Fig 3D (now 3E) - data points represent technical replicates
The antibody used in this study recognizes a slower-migrating (YAP) and a faster-migrating (TAZ) band. We have clarified which band corresponds to TAZ by the addition of an asterisk. The caption also reflects this change.
> This appears to address the wrong figures highlighted by Reviewer #2, and clarification regarding quantitation and number of replicates is required.

Author's response: Apologies for the confusion here. We will address these points here:

Fig 4A is a very technically involved experiment with many steps (ie requiring clean biochemical fractionations in particular). It is very diYicult to get all of the enrichment markers (a tubulin and Histone H3) to work without cross contamination of fractions with these cells in one experiment (nuclear/cytoplasmic, +/- Carm1 ectopic expression). Since this was essentially a descriptive experiment, while we worked on optimising the fractionation in several experiments, we only did the final experiment on the appropriately enriched fractions documenting TAZ and phospho TAZ in one biological rep (now indicate din the Fig legend). The only point that we are making from these data is that TAZ phosphorylation is greater in the cytoplasm than in the nucleus (as has been shown in multiple studies in the literature) and that CARM1 ectopic expression enhances TAZ phosphorylation in the cytoplasm. This interpretation is obvious looking at the blots. No densitometric analysis is required to come to this conclusion.

Fig 4B is an immunoprecipitation showing the interaction between TAZ and Carm1. It has been repeated multiple times and what is shown is a representative experiment confirming the mass spectrometric identification of the interaction. Densitometric quantitation of IP data is essentially meaningless since it is binary, either showing that the interaction occurs or it doesn't. The actual

© 2025. Published by The Company of Biologists under the terms of the Creative Commons Attribution License (https://creativecommons.org/licenses/by/4.0/).

levels of the proteins reflect the eYiciency of the antibodies in the IP which renders densitometric quantitation of little, if any, interest.

The point regarding Fig 3E (we think Fig3F in the recent re-submission) is that it simply supports the quantitation experiment done in what is now Fig 3E. The current Fig 3E definitively shows that there are many more Myogenin positive cells when the myogenic cells are treated with the Carm1 inhibitor (which is already documented in the literature). There is really not much need for the Myogenin western blot here which could be removed if necessary. We have indicated in the legend that N=1 for this experiment.

In terms of the asterisks in the figures. They refer to the Mr of FlagTAZ (Fig 4B) Or endogenous TAZ (Fig 4A). See comments above regarding N numbers.

Most of my comments have been addressed in a satisfactory manner. However, in response to my comment 2 about what data points mean and what the N is for each experiment, the response is not clear and at odds with the changes to the manuscript. The response states:

Fig 2F - datapoints represent independent biological replicates (luciferase)
Fig 3B - data points represent independent biological replicates(luciferase)
Fig 3D (now 3E) - data points represent technical replicates

In fact the figure legends for 2F, 3C (not 3B, this must be an error), and 3D all say the points represent technical replicates from an "individual (or "one") biological sample". What does this actually mean? Transfections were performed on three plates of the same cells in the same experiment? One plate with three assays? It is generally accepted that the number of biological replicates constitutes the N for the experiment. Unless these experiments have been performed in a way I don't understand, they therefore have N=1. N should equal at least 3.

Author's response: Lastly, we apologize that in the revised manuscript, a repeated mistake was made in the legends for Figs 2F and 3B (this, we think, was Fig 3C in the revised version). We have now corrected this as these were biological (not technical) replicates. So for each of these experiments the values of triplicate intra-experimental samples were determined and the mean of those triplicates constituted 1 biological replicate. Each experiment was repeated 3 or more times and the data points on the graphs indicate the Biological replicates (N=4 for Fig 3C, N=3 for Fig 3C). In the case of Fig 3D (now 3E) - the multiple data points (>10 for each condition) in this case do represent technical replicates of one experiment (N=1 Biological replicate). These N numbers are now correctly referred to in the Legends.

<u>Third decision letter</u>

MS ID#: jcs.263527R2

MS Title: TAZ (WWTR1) Interactome Analysis using nanotrap based affinity purification-mass spectrometry

Authors: John C. McDermott; Jonathan Kelebeev; Anastasia MacKeracher; Tetsuaki Miyake

Article Type: Tools and Resources

Dear Dr McDermott,

I am happy to tell you that your manuscript has been accepted for publication in Journal of Cell Science, pending standard publication integrity checks. It was accepted on 15 Jan 2025.

<u>Reviewer 1</u>

© 2025. Published by The Company of Biologists under the terms of the Creative Commons Attribution License (https://creativecommons.org/licenses/by/4.0/).

Advance summary and potential significance to field

Comments for the author

The authors have addressed most of the reviewers comments/suggestions, including additional text for clarification, and extra experiments/quantitation (figure 3A and Figure S2).
However, there were some instances where the reviewers comments have not yet been directly addressed and these are outlined below.

Reviewer #1, point 7:
7. Fig 3A, S2A, 4A - labels in figures indicate that the antibody recognises YAP/TAZ, although only TAZ is mentioned in the results text. There are also two bands in fig 4A - do these correspond to YAP and TAZ? If so, could authors clarify if the immunofluorescence in 3A and S2A is specific for TAZ?
RESPONSE: The antibody used in this study recognizes a slow-migrating (YAP) and a faster-migrating (TAZ) band. We have clarified which band corresponds to TAZ by the addition of an asterisk. The caption also reflects this change.
> Please address the question regarding the immunofluorescence specificity. If it recognises both YAP/TAZ, this should be clarified in the main text as well.

Reviewer #1, point 8:
8. Fig 3E - Decrease in myogenin protein levels in cells treated with CARM1 inhibitor. Actin loading also looks decreased - this should be quantitated.
RESPONSE: We did go back and quantitate the Myogenin level with and without the CARM1 inhibitor nornmalized for the Actin level. The result is shown below. We did not include this additional documentation in the manuscript since the effects of Carm1 on myogenesis is a peripheral aspect of our manuscript. Other studies have shown a general effect of Carm1 on myogenesis. Of course this is a sum of the effect on all Carm1 targets and doesn't necessarily address the TAZ/Carm1 interaction specifically.
> While the finding may be a peripheral aspect, reviewer #2 made a similar comment regarding quantitation. Authors should at least tone down their conclusion in the manuscript ("Western blot analysis confirmed a decrease in Myogenin protein levels in C2C12 cells treated with the CARM1 inhibitor compared to the DMSO control") given the quantitation is based on a single biological replicate and is not presented.

Reviewer #2, point 2B:
B. For Figures 3E, 4A, and 4B: The Western blots are all discussed in a quantitative manner but no densitometric analysis or normalization to controls was performed, nor was the number of biological replicates reported. Also, what do the asterisks in Figures 4A and 4B indicate? I initially thought this might be heavy chain but 4A is a straight WB. Again, no N value is reported.
RESPONSE:
We have now clarified issues related to this point in the related Figure captions as follows:
Fig 2F - datapoints represent independent biological replicates (luciferase)
Fig 3B - data points represent independent biological replicates(luciferase)
Fig 3D (now 3E) - data points represent technical replicates
The antibody used in this study recognizes a slower-migrating (YAP) and a faster-migrating (TAZ) band. We have clarified which band corresponds to TAZ by the addition of an asterisk. The caption also reflects this change.

© 2025. Published by The Company of Biologists under the terms of the Creative Commons Attribution License (https://creativecommons.org/licenses/by/4.0/).
